# Computational and experimental analysis of bioactive peptide linear motifs in the integrin adhesome

**DOI:** 10.1371/journal.pone.0210337

**Published:** 2019-01-28

**Authors:** Kevin T. O’Brien, Kalyan Golla, Tilen Kranjc, Darragh O’Donovan, Seamus Allen, Patricia Maguire, Jeremy C. Simpson, David O’Connell, Niamh Moran, Denis C. Shields

**Affiliations:** 1 School of Medicine, University College Dublin, Dublin, Ireland; 2 Conway Institute of Biomolecular and Biomedical Research, University College Dublin, Dublin, Ireland; 3 Molecular and Cellular Therapeutics, Royal College of Surgeons in Ireland, Dublin, Ireland; 4 School of Biology and Environment Science, University College Dublin, Dublin, Ireland; 5 School of Biomolecular and Biomedical Science, University College Dublin, Dublin, Ireland; Nanyang Technological University, SINGAPORE

## Abstract

Therapeutic modulation of protein interactions is challenging, but short linear motifs (SLiMs) represent potential targets. Focal adhesions play a central role in adhesion by linking cells to the extracellular matrix. Integrins are central to this process, and many other intracellular proteins are components of the integrin adhesome. We applied a peptide network targeting approach to explore the intracellular modulation of integrin function in platelets. Firstly, we computed a platelet-relevant integrin adhesome, inferred via homology of known platelet proteins to adhesome components. We then computationally selected peptides from the set of platelet integrin adhesome cytoplasmic and membrane adjacent protein-protein interfaces. Motifs of interest in the intracellular component of the platelet integrin adhesome were identified using a predictor of SLiMs based on analysis of protein primary amino acid sequences (SLiMPred), a predictor of strongly conserved motifs within disordered protein regions (SLiMPrints), and information from the literature regarding protein interactions in the complex. We then synthesized peptides incorporating these motifs combined with cell penetrating factors (tat peptide and palmitylation for cytoplasmic and membrane proteins respectively). We tested for the platelet activating effects of the peptides, as well as their abilities to inhibit activation. Bioactivity testing revealed a number of peptides that modulated platelet function, including those derived from α-actinin (ACTN1) and syndecan (SDC4), binding to vinculin and syntenin respectively. Both chimeric peptide experiments and peptide combination experiments failed to identify strong effects, perhaps characterizing the adhesome as relatively robust against within-adhesome synergistic perturbation. We investigated in more detail peptides targeting vinculin. Combined experimental and computational evidence suggested a model in which the positively charged tat-derived cell penetrating part of the peptide contributes to bioactivity via stabilizing charge interactions with a region of the ACTN1 negatively charged surface. We conclude that some interactions in the integrin adhesome appear to be capable of modulation by short peptides, and may aid in the identification and characterization of target sites within the complex that may be useful for therapeutic modulation.

## Introduction

Protein interactions control many key cellular processes, but are often difficult to target with compounds. Short linear motifs represent potential targets for peptides and peptidomimetics, but they often act co-operatively[1]. Integrin signaling between the states of the extracellular matrix and intracellular actin filaments[2] relies not only on direct interactors [3–7], but on large focal adhesion complexes characterized as an ‘adhesome’ comprising over 180 proteins and at least 742 interactions [2,8]. This adhesome plays a key role in platelet signaling, as integrin activation is the final common step that leads to platelet activation following stimulation via numerous pathways[9]. Platelet activation is an important therapeutic target for the treatment of coronary artery disease[10], and integrin signaling is also important in Multiple Sclerosis[11]. Platelet integrins[12] are targeted extracellularly via small molecule, peptidomimetic and antibody therapeutics[13], but given their “inside-out” signaling mechanisms, it is also of interest to modulate the multiplicity of different adhesome conformations, targeting various intracellular adhesome interfaces.

The adhesome network superficially resembles a robust system that may be resistant to therapeutic modulation, in the sense that many of its components have multiple interactions, permitting compensation for the knockout of any factor by other interacting components. Adhesome proteins that are embryonic lethal (see S1 Table) tend to have many interactors (i.e. are “hub” proteins, such as integrin, paxillin, Grb2 and FAK), while those with fewer interactors such as tensin, vimentin and IAP have less deleterious consequences [8][14]. Knockout of the highly connected Src is not lethal, perhaps because of compensation by the homologous kinases Lyn and Fyn[8]. However, knockout and over-expression studies of proteins ignore their many separate domains and motifs, which may have synergistic, antagonistic or independent effects. To develop potential therapeutic strategies, more reagents are needed which specifically target particular interaction interfaces within the adhesome, including short linear peptide motifs, and there is a need to map their co-operative properties.

We constructed a virtual platelet integrin adhesome and screened computationally across the network of candidate proteins to discover motifs likely to play a role in protein interactions. We investigated their effects on integrin activation and its inhibition, delivering synthetic peptides inside the cell individually, in chimeric combinations, and as combinations of independent peptides.

## Results

### Integration of integrin adhesome and platelet expression data to generate a platelet-relevant integrin adhesome

Here, we considered the integrin adhesome as a set of interacting short linear motifs. To generate a set of proteins that make up the platelet integrin adhesome, we first created a dataset describing the set of proteins present in platelets. This dataset was then used to find platelet-expressed members and homologues of the components of the curated integrin adhesome [2]. This platelet proteome dataset was assembled from three sources: (A) Merging a number of published proteomics datasets [6]–[10]. Generally each dataset targeted a different fraction of the proteome, thus providing a more complete view of the entire proteome. (B) The Normal Clinical Tissue Alliance dataset, published on GPMDB [15] contains proteomics data which indicates which proteins are frequently found in different tissue types and biological samples. (C) A third dataset containing proteins from an integrin pull-down experiment listing proteins that are likely to associate with integrin αIIbβ3 was obtained ([3]; NM unpublished data). All three of these datasets were merged to create the platelet proteome. Uniprot mappings were updated using the Uniprot retrieval tool and proteins were then matched by ID.

**Fig 1:**
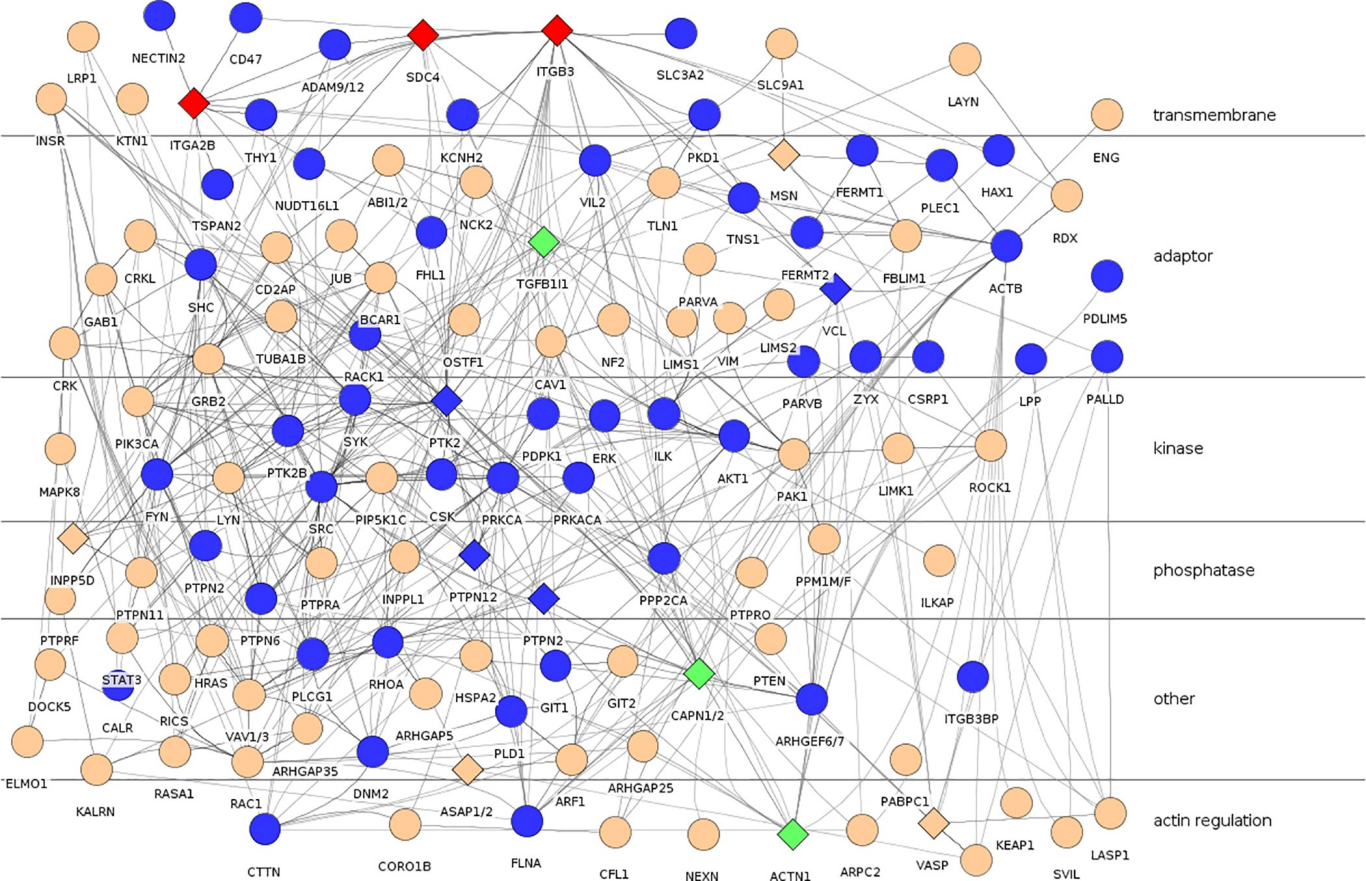
Platelet integrin adhesome highlighting proteins containing bioactive peptides. Diamonds represent proteins from which peptides were derived. Red: proteins from which active palmitylated peptides were derived. Green: proteins from which active tat peptides were derived. Circles: other proteins. Edges represent interactions annotated in the integrin adhesome [2], and inferred homologous interactions.

This merged dataset was then used to select protein members of the platelet integrin adhesome. Each protein in the integrin adhesome was mapped to platelet proteome proteins, using BLASTP 2.2.25+ with default settings. Results were then filtered (query sequence coverage > = 50%, sequence identity > = 25%, E-value < = 1.10^−8^). Proteins which did not match perfectly were then subject to a manual literature and platelet web [16] search to determine their presence in platelets as described in the methods section. The result was a smaller network of putative platelet adhesome proteins (Fig 1; S1 Data).

Of the 189 integrin adhesome proteins that were matched to a platelet proteome drawn from three described platelet protein sets, a total of 141 were present in platelets (see first sheet of S1 Data). Manual curation of the homology search results versus the platelet proteome was used to supplement the platelet adhesome with an additional 11 platelet proteins (PDLIM5, TSPAN2, ADAM9, CD2AP, ARHGAP25, FHL1, PLCG2, NECTIN2, DOCK5, KALRN and AKT2). These were homologous to integrin adhesome proteins that were not themselves present in the platelet proteome, and are therefore good candidates to fulfil their role in the platelet. Thus, a total of 152 proteins were identified as likely platelet integrin adhesome components for further peptide searching. 13 of these were integrin proteins, but we wanted to focus our efforts on the primary platelet integrins αIIb and β3, so we excluded the other 11 integrin proteins (ITGAV, ITGA5, ITGA2, ITGA6, ITGA3, ITGAX, ITGB6, ITGB2, ITGB1, ITGB5 and ITGB7) from our peptide selection, yielding a search set of 141 proteins.

We selected peptides from those proteins likely to act at interfaces between nodes in this network, based on known protein-protein interactions of residues within the peptide (Table 1), combined with computational predictions within the disordered regions of proteins, in which short linear motifs (SLiMS) are enriched[17]. Known interactions were identified by a reading of the available literature, with a focus on those where the regions of interaction were identified. Thus, while some of the peptides were identified purely computationally, others were supplemented by a reading of the literature, and in many cases there was overlap between the two groups (Table 1), with the final choice of peptides drawing on the evidence from both sources. The computational prediction methods used were SLiMPred[18], a machine learning predictor of SLiMs which relies on the primary amino acid sequence of the protein, and SLiMPrints[19], which identifies motifs based on their enriched local evolutionary conservation in orthologues.

**Table 1:** Features of selected peptides.

Peptide Name	Peptide Choice Rationale	Gene name	Sequence	Activation	Inhibition	Phospho	Average SLiMPred	Max SLiMPred	SLiMPrints
pal-ITGA2B_JM	Interaction[21]	ITGA2B	pal-KVGFFKR-(NH2)	17*	50	R	0.23	0.3	-
pal-ITGA2B_tail	Interaction[23]	ITGA2B	pal-LEEDDEEGE	2	52		0.13	0.43	-
pal-ITGB3_tail	Interaction[24]	ITGB3	pal-TNIT**Y**RGT	3	71		0.01	0.04	-
pal-ITGB3_JM	Interaction[25]	ITGB3	pal-KLLITIHDRKE-(NH2)	4	73		0.03	0.06	-
pal-ITGB3_middle1	Interaction[26]	ITGB3	pal-FAKFEEERAR-(NH2)	0	45		0.04	0.2	-
pal-ITGB3_middle2	Interaction[24]	ITGB3	pal-NNPL**Y**KEA-(NH2)	1	74		0.16	0.41	0.6
pal-SDC4_JM	SLiMPred; Interaction[27]	SDC4	pal-RMKKKDEGSYD-(NH2)	0	4*		0.41	0.64	-
pal-SDC4_middle	Interaction[27]	SDC4	pal-LGKKPIYKK-(NH2)	0	3*		0.13	0.25	-
pal-SDC4_tail	SLiMPred; Interaction[27]	SDC4	pal-APTNEF**Y**A	7	34*		0.51	0.71	-
Tat	Interaction[24]		tat-(NH2)	3	73		0.07	0.16	
TGFB1I1_LD-tat	SLiMPrints; SLiMPred; Interaction[28]	TGFB1I1	TLELDRLMASLSDFRVQNHLP-tat-(NH2)	3	66	R	0.15	0.57	0.004
tat-ACTN1_VBS	Interaction[29]	ACTN1	tat-WEQLLTTIARTINEVENQI-(NH2)	66*	61*	R	0.04	0.22	-
tat-VCL	SLiMPred, Interaction[30]	VCL	tat-EPDFPPPPPDLE-(NH2)	4	70		0.58	0.88	0.14
tat-VASP	SLiMPrints; Interaction[31]	VASP	tat-AGAKLRKV**S**KQE-(NH2)	3	74		0.22	0.49	0.01
tat-CAST_1	Interaction[32]	CAST	tat-DPMSSTYIEELGKREVTIPPKYRELLA-(NH2)	3	72	A	0.06	0.2	-
tat-CAST_2	SLiMPred; Interaction[33]	CAST	tat-SKPIGPDDAIDALSSDFTS-(NH2)	3	74		0.18	0.59	-
tat-MSN	Interaction[34]	MSN	tat-GRDKYK**T**LRQIRQGNTKQRIDEFE**S**M	3	73		0.1	0.41	0.07
tat-NHERF1	SLiMPrints; Interaction[35]	NHERF1	tat-KRAPQMDWSKKNELFSNL	3	70		0.18	0.44	0.04
tat-FAK_1	Interaction[36]	PTK2/ FAK1	tat-EGERALPSIPKLAN-(NH2)	4	72		0.07	0.23	-
tat-FAK_2	SLiMPrints; Interaction[36]	PTK2/ FAK1	tat-SVSETDDp**Y**AEIIDE-(NH2)	3	71		0.06	0.17	0.001
AMAP1-tat	SLiMPrints; Interaction[37]	ASAP1/ DDEF2	SSTLSKKRPPPPPPGHKRTL**S**D-tat-(NH2)	4	72		0.25	0.49	0.002
PTPN1-tat	SLiMPrints	PTPN1	GIESMSQDTEVRSRVVGGS-tat	3	77		0.14	0.37	<0.00001
INPP5D-tat	SLiMPred; SLiMPrints	INPP5D	KLSQLTSLLSSIE-tat	4	74		0.46	0.9	0.01
PTPN12-tat	SLiMPred; SLiMPrints	PTPN12	NSDTPPRPDRLPL-tat	3	75		0.48	0.89	0.003

Identification of peptides modulating platelet aggregation. Pal: peptides are N-terminally palmitylated (peptides tested at 20μM). tat: addition to the peptide of the cell-penetrating peptide sequence GRKKRRQRRRPPQ at the indicated terminus (peptides tested at 50μM). Activation: platelet aggregation induced in resting platelets after 6 minutes incubation with peptides, quantified as optical density using an aggregometer, n> = 3. Inhibition: platelet aggregation induced by 4μM TRAP in platelets pre-incubated with peptide. Phospho: Phosphorylation changes associated with peptide treatment, R: in resting platelets, A: after TRAP activation. Underlined bold residues represent known phosphorylation sites: the tat-FAK_2 peptide was synthesized in its phosphorylated form (indicated by Y preceded with a lower case p). Cell delivery mechanisms are indicated as pal and tat. JM = juxtamembrane. Peptide Choice Rationale: “Interaction” indicates that there is an experimental evidence that residues in this peptide are involved in adhesome protein-protein interactions; “SLiMPred” indicates a maximum score for a residue in the peptide > 0.5; and “SLiMPrints” indicates a significance (Sig) score of less than 0.05 for a motif within or overlapping the peptide.

*: significantly (p≤0.05; see S1 Fig) higher aggregation than resting platelets (activation column) or lower than TRAP-activated platelets (inhibition column).

### Screening for peptide effects on platelet activation and inhibition

Prioritized peptides which were derived from the cytoplasmic regions of integrin and syndecan transmembrane proteins were synthesised with a palmitylation at their N-terminus. This membrane-anchoring peptide delivery approach not only delivers the peptide to the cell, but is also intended to target the peptide closer to its natural context[20–22] by replacing the transmembrane region that anchors the peptide with a lipid group. For synthesized peptides from other proteins, a cell-penetrating tat peptide was added to a peptide terminus.

We identified in Table 1 a number of peptides that either stimulated platelet aggregation, or inhibited platelets from being activated by the thrombin receptor activating TRAP peptide[38]. Fig 1 shows the proteins from which these peptides were derived, in the context of the platelet adhesome.

Platelet activation was inhibited by three peptides spanning the short cytoplasmic tail of syndecan-4 (SDC4) (Table 1). Each peptide has a different candidate binding partner (S2 Table), with SDC4_JM in the juxtamembrane region binding syndesmos [39]; SDC4_middle binding to PKCα[40], and SDC4_tail region binding to syntenin via its PDZ binding motif [41]. Scrambled versions of the positively charged pal-SDC4_JM and pal-SDC4_middle peptides also inhibited platelet aggregation (S2 Fig), implying that their action may depend on amino acid composition, increasing the likelihood of off-target effects unrelated to SDC4 interactions. Surface plasmon resonance (SPR) indicated that a slightly extended version of the SDC4_tail peptide had higher affinity for syntenin than a scrambled version (S3 Fig) which did not inhibit platelet activation (S2 Fig), consistent with specific interaction of the peptide with its binding partner as an explanation for its effects on platelet function.

As expected, platelet activation by TRAP increased tyrosine phosphorylation of many proteins (S4 Fig). Tat-CAST1 increased the phosphorylation intensity of the TRAP activated platelets, despite having no effect on phosphorylation in resting platelets (p = 0.03, S4C Fig). CAST1 is a known inhibitor of Calpain, a protease that cleaves many proteins involved in phosphorylation.

### Chimeric and combination peptide effects

The integrin adhesome is likely to be a relatively flexible complex, adopting different conformations in different structural and signaling contexts. Accordingly, chimeric peptides containing multiple motifs could induce novel protein interactions that alter activity. Chimeric peptides were generated that joined motifs of the following classes: integrin_U_integrin, integrin_U_actinin, integrin_U_TGFB1I1, integrin_U_syndecan, and syndecan_U_syndecan, where a subscripted U indicates a chimerization of two active peptides. Those involving integrin peptides did not appear to be strikingly more potent inhibitors or activators (S3 Table). One of the strongest inhibitory syndecan_U_syndecan chimeras spanned the middle (variable) and tail regions of SDC4 (S5 Fig); notably this is a natural peptide, bringing the two regions together in their natural sequence. This may potentiate the chimera binding to multiple partners simultaneously, since the variable region binds PKC and PIP2, while the C-terminal tail region binds syntenin.

**Fig 2:**
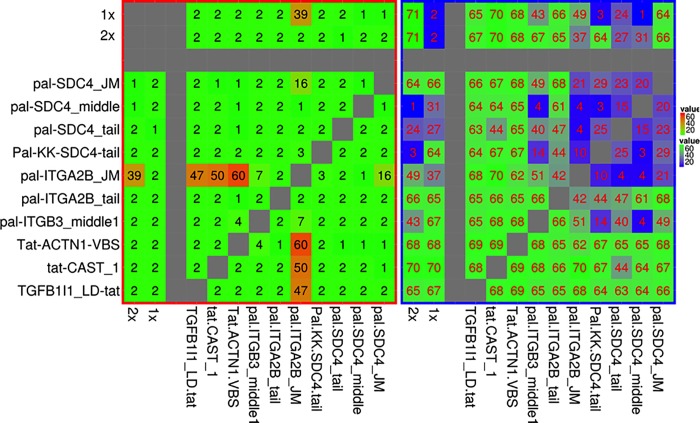
To explore potential synergy of peptides of interest, combinations of peptides were investigated (same result above and below diagonal). Left: platelet aggregation induced in resting platelets after 6 minutes incubation with peptides, quantified as optical density using an aggregometer, n = 3 (lowest unactivated values in green, highest activated combinations in orange/red). Right: inhibition of TRAP (2 μM) activation of platelets after pre-incubation of the platelets for 6 minutes with the peptides, n = 3 (full activation is indicated in bright green, greatest inhibition of activation is shown in dark blue). Peptide concentrations: 4 μM for pal-peptides and 10 μM for tat-peptides; 1X: peptide alone at that concentration; 2X: peptide alone, but at twice that concentration. See S7 and S8 Figs for more details.

Another approach to explore the cooperation of SLiMs in the adhesome is to simultaneously target multiple interfaces with combinations of separate peptides, which are not sterically limited by the linkages of chimeric peptides. We considered all pairwise combinations of ten peptides (S4 and S5 Tables), investigating additive effects, where the two peptides in combination had a greater effect than either peptide alone at the same dose, and synergistic effects, where the peptides in combination had a greater effect than either of the two peptides alone at a doubled concentration[42], meeting a strict definition of true synergy[43]. For platelet activation, the pal-ITGA2B_tail peptide combined additively with the three tat-ligated peptides tat_CAST_1, TGFB1I1_LD_tat and tat_ACTN1_VBS (Fig 2), and synergistically with tat_ACTN1_VBS (combination more activatory than a double concentration of either tat_ACTN1_VBS, p = 0.03; or pal-ITGA2B_JM, p = 0.04). While still significant when the experiment was repeated (p = 0.04, p = 0.05 versus double doses, S6 Fig), we noted that these effects could be more simply explained by synergy of pal-ITGA2B_tail with tat peptide alone, even though tat on its own had no activatory effect. The positively charged peptide sequence of the tat peptide may synergize with pal_ITGA2B_JM activation by potentiating its delivery through the membrane, or it may act on independent targets in the platelet.

### Vinculin binding peptide bioactivity

**Fig 3:**
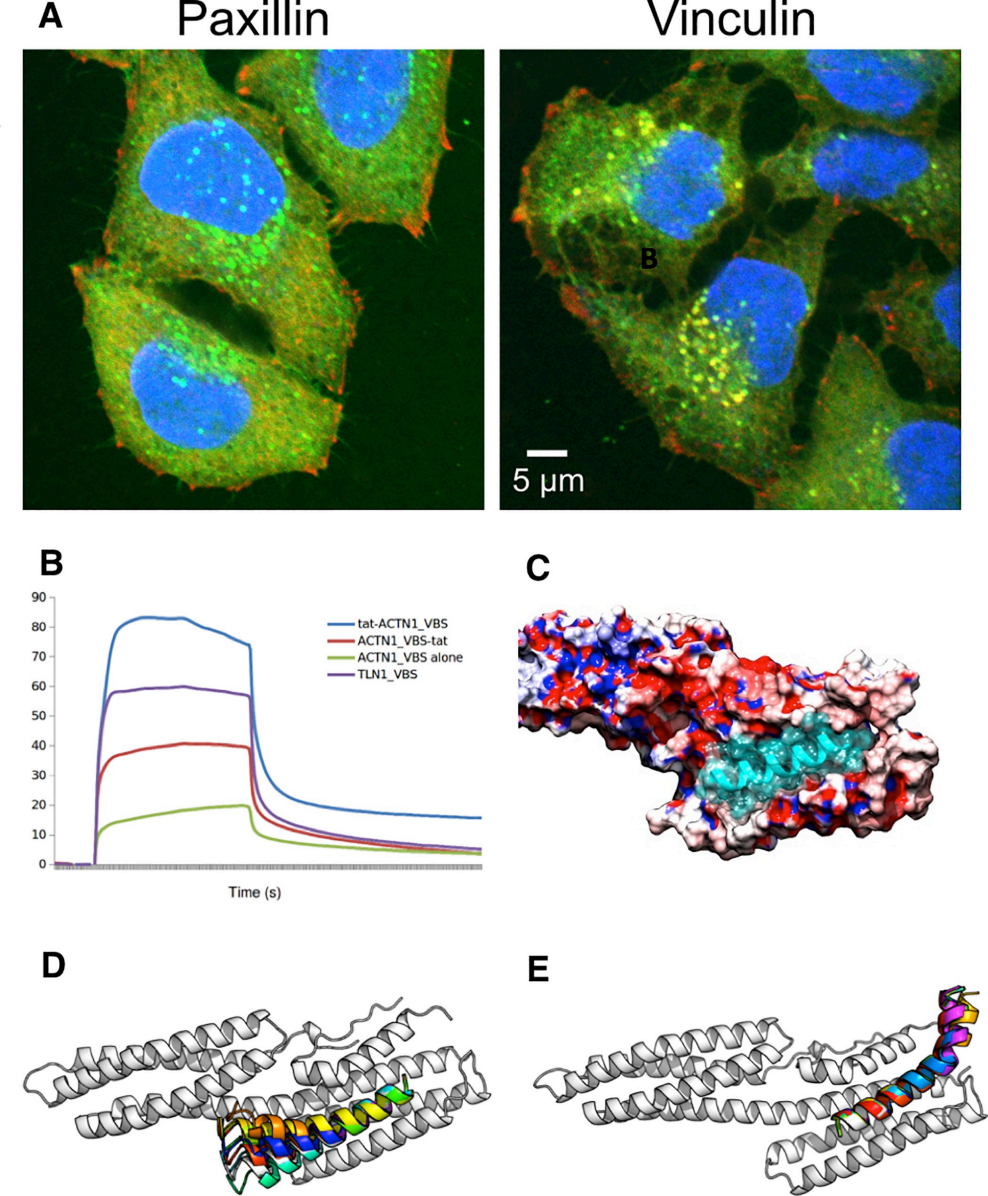
Specificity of vinculin binding region peptides. **(A)** HeLa cells treated with 5FAM-labeled tat-ACTN1-VBS peptide and immunostained with either vinculin (right) or paxillin (left) antibody. Blue: nuclear stain; green: tat-ACTN1-VBS peptide, conjugated with the 5FAM fluorophore at the N terminus; red: paxillin or vinculin staining, as labeled; yellow: co-localization of protein and peptide. Full results in S10 Fig. **(B)** Surface plasmon resonance sensorgrams for serial peptide injections at 1 mM onto immobilised vinculin on a flow cell of a CM5 sensor chip. Relative binding affinity tat-ACTN1_VBS > TLN1_VBS-tat > ACTN1_VBS-tat > ACTN1_VBS. **(C)** Electrostatics of the vinculin surface: Electrostatic surface showing the active site of the alpha actinin peptide ACTN1-VBS (not including tat) binding to vinculin (PDB entry 1YDI). The region in which the positively charged tat sequence is likely to bind is a negatively charged (red) region of vinculin. Positively charged regions are shown in blue. **(D)** FlexPepDock [28] was used to predict the binding poses of the ACTN1-VBS-tat peptide where the tat peptide is located at the peptide C-terminus, to the PDB entry 1YDI, contrasting alternative termini for coupling with tat. **(E)** As for (D), but with the more stably binding tat-ACTN1-VBS peptide, in which the tat region at the N-terminus of the peptide (top right of image) appears to have been stabilized in comparison with (D), via interactions of the positively charged tat peptide with a negatively charged surface region of vinculin.

We further investigated the binding of vinculin-binding peptides. Tat-ACTN1_VBS co-localized with vinculin in HeLa cells, consistent with a direct action on vinculin, and did not co-localize with a control adhesome protein paxillin, a homologue of the platelet’s TGFB1I1 (Fig 3A). Co-localization was restricted to intracellular puncta, with peptide-free vinculin concentrated at points resembling membrane focal adhesions. This colocalization is consistent with a model in which the peptide primarily binds intracellular vinculin. Tat peptide alone failed to localize with vinculin and was spread more evenly throughout the cell (S9 Fig). Vinculin present in focal adhesions may be tightly bound to ACTN1 and other proteins, and therefore not available for binding with peptide. Since vinculin has roles in both integrin activation and inhibition[14], the co-localization of peptide with vinculin seen here could be consistent with a damping of its potential inhibitory effects, but may act through a number of potential mechanisms.

Platelet aggregation was activated by the tat-delivered peptide, tat-ACTN1_VBS, which was derived from the vinculin-binding region of α-actinin. Locating the tat peptide at the C-terminus (ACTN1_VBS-tat) resulted in a weaker platelet activating effect than the N-terminal attachment of the tat-ACTN1_VBS peptide (S10 Fig). The dissociation of tat-ACTN1_VBS from immobilized vinculin in a Surface Plasmon Resonance (SPR) experiment indicated that this peptide indeed bound with relatively higher affinity than the peptide with tat at the C-terminus (ACTN1_VBS_tat), or ACTN1_VBS peptide lacking the tat (Fig 3B). Different peptides are known to bind vinculin in opposite orientations. The TLN1 binding site binds in the opposite N to C terminal orientation to ACTN1, justifying the choice of the tat ligation at the C terminus of the peptide, and it showed a stronger binding affinity than ACT1_VBS-tat, consistent with the computational model of charge interactions.

A computational model using FlexPepDock[28] predicted that tat-ACTN1_VBS indeed formed a more stable interaction, with the tat region in contact with negatively charged regions of the vinculin surface (Fig 3). In this model, the initial pose of the VBS peptide region was determined by the peptide pose in the complex of peptide and vinculin (Fig 3C), and this alpha helical pose was maintained during the modelling, while the tat peptide conformation was computed. The conformation of the C-terminally attached tat was unstable (Fig 3D), while that of the N-terminally attached tat was stable (Fig 3E). The factors which stabilize one tat orientation versus the other may include a number of possible interactions in addition to the electrostatic interactions, and it is likely that the interactions in the central VBS peptide region are dominated by van der Waal interactions between the hydrophobic faces of the peptide and vinculin, as previously shown[29].

## Discussion

**Fig 4:**
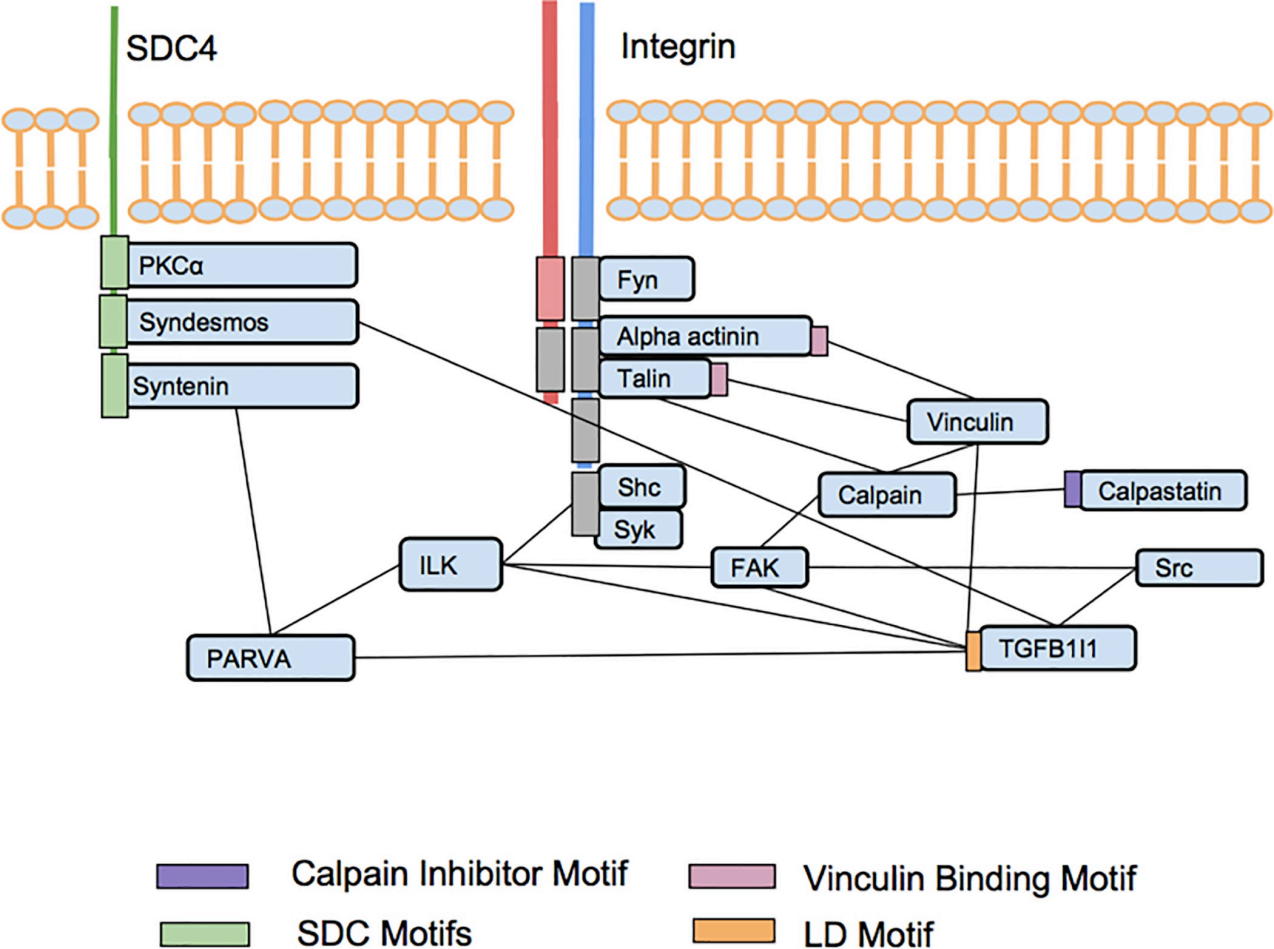
Integrin adhesome components with peptide interfaces modulated by synthetic peptides. Top of image: extracellular. Bottom of image: intracellular. Red and blue lines spanning the membrane: integrin subunits. Green line: SDC4 protein. Light blue rectangles: proteins. Other colored rectangles indicate motif regions in the proteins mimicked by synthesized peptides (gray are inactive). Black straight lines: protein-protein interactions.

Our screen of bioactive peptide action and interaction in the platelet adhesome identified activators and inhibitors of platelet aggregation, which may represent interfaces of interest for perturbation (Fig 4). The integrin adhesome appeared relatively robust against the challenge presented by combinations of agents. While the lack of synergy among covalently joined chimeric peptides could arise from particular steric hindrance or distance constraints within the adhesome, the lack of very marked synergies among combined separate peptides of the adhesome suggest that robustness versus synergistic effects may be a feature of signalling within the adhesome network. While marked synergies were not shown, we observed a number of clear additive effects, and those between integrin and syndecan intracellular peptides are consistent with prior evidence that their proteins synergize functionally[27]. The integrin adhesome is critical to the final step of platelet aggregation, and may interact via its different components with other pathways. It is possible that the adhesome may have evolved to exaggerate particular responses to particular combinations of external stimuli via different receptors, but to dampen synergies within the complex.

Identification of peptides of interest relied firstly on computational predictions, combined with literature evidence. SLiMPred relies on a neural network trained on known short motifs that bind to peptide binding domains of proteins, while SLiMPrints identifies conserved motifs embedded within more rapidly evolving regions of disordered tracts of proteins. While we originally set out to rely entirely on computational predictions from the primary sequence of the interactors alone, investigation of the literature for the most interesting peptides proposed by computational analysis revealed that useful insights into peptides of interest could also be gleaned from the literature, by identifying known interaction interfaces between proteins, based on structural and other studies. Among the eight peptides with literature evidence of involvement in interaction surfaces that lay in disordered regions permitting SLiMPrints score calculation, five had significant SLiMPrints scores (below 0.05; Table 1). This enrichment supported the use of SLiMPrints score alone in some other tested peptides.

Of the 21 tested peptides selected with literature evidence of interaction, one third showed bioactivity (Table 1); three of the seven selected with SLiMPred evidence showed bioactivity; and one of the eight selected peptides with SLiMPrints evidence showed bioactivity. SLiMPrints is best suited to the detection of bioactivities within the context of long extended tracts of disorder, and protein interactions within the integrin adhesome appear more dominated by ordered interaction surfaces such as alpha-helices. In predicting and designing potential bioactive peptides for experimental screening, the choice of computational tools and of literature surveys of protein interaction surfaces is likely to be determined in part by the nature of the complexes under study. However, such choices may only become apparent to the researcher after spending a considerable amount of time surveying the literature and inspecting the distribution of disordered and ordered tracts in different proteins within the complex, and accordingly, a multi-pronged approach selecting predicted bioactives using a number of computational and literature-based strategies may well be a useful approach.

There are a number of important caveats in a study such as this. Firstly, the peptides may not enter the cells and encounter the integrin adhesome at the concentrations that they are provided in solution outside the platelets: depending on their sequences, aggregation of peptides, along with differential efficiencies of membrane crossing, compartmentalization, trafficking and degradation within the cell, can lead to false negatives. Nevertheless, the general evidence is that palymitylated [44] and tat-associated peptides can enter the cell. While negative charge seen on some peptides may militate somewhat against cell entry, many peptides are likely to enter at some degree of efficiency, and peptides with a net negative charge in their non-tat section are included amongst the active peptides in our screen (Table 1). Visualisation of localization in HeLa cells of one of the active peptides from ACTN1 (Fig 3) is consistent with the main peptide pool migrating to an intracellular body, where vinculin is also accumulating, rather than accumulating at the target adhesome site on the membrane; it is difficult to visualize protein localization in subcellular regions of platelets, but it is possible that the peptides may be inducing or following vinculin re-localisation under different conditions. While a comprehensive analysis of the localization of peptides using fluorescent labelling would be of great interest, it was beyond the scope of this present study.

The identification of peptide effects among scrambled peptides is a cause for concern. While we had hoped that the synergy analysis would reveal activities at much lower peptide concentrations, where the likelihood of off-target effects and effects by scrambled peptides at those matching concentrations should be reduced, the set of synergistic interactions we explored failed to identify peptide activities at very low doses in the context of synergy. A wider analysis of synergy, using not only peptides but also compounds with potent effects, may be required in order to be successful with this strategy. Other strategies to overcome the likely off-target effects indicated by scrambled peptide activities include analysis with physically constrained peptides, which may adopt less promiscuous poses and thus interact with fewer targets. Such peptides are more complex to synthesise, and typically such a strategy requires structural knowledge of the peptide target interface to direct the peptide design.

The modest additive synergistic effects noted in this study could be of some interest, but must be interpreted in the light of certain scrambled peptide controls showing effects, which diluted the strength of these observations. In addition, the tat synergism with the pal-ITGA2B_tail peptide suggested the possibility of some non-specific effects of the tat peptide when in synergy with other peptides, regardless of its cargo.

Peptide screens need to present the peptide ideally as far as possible in a context that matches that in which they may occur biologically. Tat peptides may promote or discourage binding to the peptides interaction site, and palmitylation may encourage the correct binding mode, or sterically inhibit it by bringing the peptide too close to the membrane. Indeed, our SPR findings indicated a better binding of the more extended SDC4_tail peptide to syntenin, potentially consistent with some steric hindrance of the shorter peptide.

The computational tools SLiMPred and SLiMPrints enabled two broad strategies for motif discovery in a protein, the first focused on sequence properties and the second on evolutionary conservation. There are many other tools that can perform similar tasks to these. Anchor [45] seeks to identify interaction-like regions that are much longer than those targeted by SLiMPred, but we were interested in shorter regions that are more amenable to experimental perturbation. Methods exist which can explicitly take into account the network structure of a protein interaction complex, to identify motifs over-enriched in common interactors with a given domain, such as SLiMFinder [46] and Dilimot [47]. These have relatively little power in a smaller network, where there are relatively few recurrences of protein domain interaction motifs in non-homologous proteins. Simple regular expression-based searches based on known SLiM definitions [48] represent an alternative approach, and it would be interesting to know whether such alternative strategies could yield more potent modifying peptides of the system. Simpler strategies of relying on alpha-helical [49–51], disorder [52] or surface prediction may also potentially prove a more efficient means of enriching for bioactive peptides in a complex such as the integrin adhesome.

The small platelet cell has over 80,000 integrin dimers per cell [53], and relatively high peptide concentrations were needed to modulate integrin signaling. However, there is a good prospect of developing more potently binding peptides or peptidomimetics: while many human SLiMs have evolved with what appears to be deliberately modest affinity[54], pathogen peptides, including one targeting the intracellular regions of the adhesome[55], can bind with much higher affinity[54]. Future analyses of synergy within the integrin adhesome and between it and other signaling pathways may well require investigating combinations of peptides, peptidomimetic, and other small molecule activators and inhibitors. This work highlights the value of computational and literature evaluation when screening for lead peptide modulators of protein interactions derived from human proteins.

## Materials and methods

### Ethics statement

This work was approved by the IRB Committee of the Royal College of Surgeons in Ireland, approval for the use of blood samples from donors for platelet function analysis.

### Platelet proteome

A dataset of proteins found in platelets was assembled from three different sources:

A). A collection of published proteomics datasets [6]–[10] were merged to create a literature based platelet proteome.

B). The Normal Clinical Tissue Alliance dataset, published on GPMDB[15] contains proteomics data which indicates which proteins are frequently found in different tissue types and biological samples.

C). An integrin pull-down experiment listing proteins that are likely to associate with integrin αIIbβ3 ([3]; NM unpublished data).

Uniprot IDs were updated with the Uniprot retrieval tool and datasets were then merged via uniport ID.

The platelet proteome merged from these three resources is presented in S1 Data (an excel format .xlsx workbook).

### Platelet adhesome

The adhesome proteins and interactions were defined from literature[2] and each node in the network was mapped to a suitable platelets protein. A subsequent adhesome data resource [56] was not available at the time of peptide design, and so was not included in the study. Each protein in the integrin adhesome was mapped to proteins from the platelet proteome. Adhesome proteins that matched exactly were considered common to multiple cell types and added to the platelet adhesome dataset. Platelet proteome nearest homologues of integrin adhesome proteins were identified using BLASTP 2.2.25+ with default settings. Results were then filtered to choose matches with a query sequence coverage > = 50%, a sequence identity > = 25%, and an E-value < = 1.10^−8^. They were then investigated in literature and the plateletweb database, and added to the dataset if there was evidence to support their presence in platelets. While several integrins were found within the dataset, we restricted our dataset to only include the major integrin αIIbβ3 proteins for SLiM analysis.

### Antibodies and concentrations

Phosphotyrosine western blots were performed using anti-phosphotyrosine, clone 4G10 (Millipore, Ireland), antibody with a dilution of 1 in 1000. Paxillin (610619, clone 165, from BD Biosciences) and vinculin (ab18058, clone SPM227, Abcam, UK) primary antibodies were used in microscopy experiments with dilution of 1/100. All general materials were purchased from Sigma Aldrich, Ireland. Thrombin Receptor Activating Peptide (TRAP) was purchased from BACHEM, Switzerland. All platelet aggregation materials were purchased from BIO/DATA Corporation, UK.

### Peptide synthesis

Peptides were synthesized by Peptide2 (www.peptide2.com) and verified by HPLC and mass spectrometry. Purities greater than 80% were required for all peptides. Peptides used in SPR experiments were of at least 95% purity. Modifications for particular peptides were conjugated with a palmityl group or with the 5FAM fluorophore at the N-terminus, and addition of a tat peptide sequence at the terminus indicated for each peptide.

### Peptide preparation

Peptides were stored in powder form at -80°C. Prior to use, they were left at room temperature for one hour. They were then mixed with a specific volume of deionised water to bring them to a 1mM concentration and were then aliquoted and frozen at -80°C. They were thawed a single time before use and discarded after an experiment. Peptides which were not soluble in deionised water (pal-APTNEFYA, pal-LEEDDEEGE, pal-TNITYRGT and pal-KLLITIHDRKE) were dissolved in a 10% DMSO and 90% deionised water solution (leading to a final concentration of 0.2% DMSO when combined with platelets, see below). These peptides were further diluted during platelet aggregations.

### Washed platelet preparation

Blood was drawn from healthy volunteers into a syringe containing a final volume of 15% Acid Citrate Dextrose (ACD). The blood/ACD mixture was then aliquoted into 15 ml Cellstar tubes, at volumes of 5ml per tube, and centrifuged at 150 rcf for 10 minutes to obtain platelet rich plasma (PRP). PRP was then transferred to a 50ml tube using a pipette. The pH was then adjusted to 6.5. Prostaglandin (PGE1) was then added with a final concentration of 1μM. The PRP was then centrifuged at 750 rcf. After centrifugation the platelet poor plasma (PPP) was discarded and the pellet was resuspended in buffer A (6 mM dextrose, 130 mM NaCl, 9 mM NaHCO3, 10 mM sodium citrate, 10 mM Tris base, 3 mM KCl, 0.81 mM KH2PO4 and 0.9 mM MgCl26H2O, pH 7.4). A platelet concentration of 3x10^5^/μl or 1x10^6^/μl was obtained by dilution depending on the experiment in which they were used. Platelets were left to rest for 45 minutes and supplemented with 1.8mM calcium chloride prior to use.

### Platelet aggregation

A platelet concentration of 3x10^5^ /μl was used when performing standard aggregations and a concentration of 1x10^6^ /μl was used for sample preparation for western blot analysis. The extent of platelet aggregation was measured by quantifying the optical density of the samples using the Bio-Data PAP-8 aggregometer (platelet aggregation profiler, Horsham, PA, USA). A temperature of 37°C was maintained and samples were stirred using magnets spinning at 1100 rpm. 200 μl of washed platelets was added to each aggregation tube. Different volumes of buffer, peptide and TRAP were added to achieve the desired concentrations. Peptide/buffer was added to each well after two minutes. TRAP, with a final concentration depending on the experiment, was added 6 minutes later and data was collected for an additional 7 minutes.

### Sample preparation

After the aggregation step, tubes were immediately placed on ice. 27 μl of lysis buffer was then added and the tube was vortexed vigorously. Lysates were left on ice for one hour and vortexed frequently. Proteins were then centrifuged at 14,000 rcf for 10 minutes to remove debris. Samples were then mixed with 2x sample buffer. 50 μl each sample was added to 50 μl of sample buffer. The protein was then denatured by heating to 95°C for 5 minutes. 10 μl of lysate was kept for protein quantification. All samples and lysates were frozen at -80°C.

### Protein quantification

The Bradford assay was then performed on the lysates to determine protein concentration. Protein concentrations were measured using the Bio-Rad DC Protein Assay. A calibration was created using samples containing a known concentration of bovine serum albumin. These were prepared in advance by sequential dilutions with final concentrations of 1.5mg/ml, 1mg/ml, 0.8mg/ml, 0.6mg/ml, 0.4mg/ml, 0.2mg/ml, 0mg/ml. Samples were then dispensed into a 96 well plate and the Bio-Rad DC reagents were added as described in the protocol. Samples were left to rest for 15 minutes and were then read using 690 0.15 mode on the Wallac plate reader. A standard curve was calculated from the standard samples of known concentration and the y = ax+b equation was used to determine the concentration of the other samples.

### Western blotting

Sodium Dodecyl Sulfate Polyacrylamide Gel Electrophoresis (SDS-PAGE) was performed to separate proteins by molecular weight. Polyacrylamide gels were prepared (see Reagents section). Samples or molecular markers were loaded in volumes to give 20 μg of protein. The gels were then run for approximately 80 minutes or until the samples reached the end of the gel. The proteins were then transferred to polyvinylidene difluoride (PVDF) membranes using a Bio-Rad Wet Blot Transfer Cell apparatus. During the transfer the membranes were submerged in 1x transfer buffer (10% 10x transfer buffer, 70% deionised water and 20% methanol). Transfer was performed either overnight at 40 volts at 4°C, or at 100 volts for 1 hour cooled with an ice block. After the transfer the membranes were blocked for 1 hour using 3% bovine albumin serum (BSA) dissolved in TBS-T. Membranes were incubated with 4G10 antibody with a 1:1000 dilution. Unbound primary antibody was washed off with TBS-T three times for 5 minutes each. Membranes were then incubated with anti-mouse IgG secondary antibody (Millipore) with a dilution of 1:30,000. Membranes were submerged in chemiluminescence ECL solution (Thermo Scientific). They were then measured using the western blot developer UVP. One image was taken every 80 seconds for 40 minutes. Numeric data was extracted from selected blots using ImageJ.

### Microscopy

HeLa cells were seeded on a 24-well plate with inserted coverslips at a density of 30000 cells/well. After 24h the cells were treated with 15 μM peptide for the desired amount of time (5, 10, 15 minutes). Cells were then fixed in 3% paraformaldehyde (PFA, Sigma) for 20 minutes and permeabilized with 0.1% Triton X-100 (Sigma) for 5 minutes at room temperature. Cells were then washed 3 times with PBS and stained with antibodies. Staining with primary antibody (paxillin/vinculin (Abcam)) was performed for 30 minutes, followed by three PBS washes and secondary staining using goat anti-mouse Alexa 568 antibody (Life Technologies) for 30 minutes. Then the nuclei were stained with 0.2 μg/ml Hoechst33342, the coverslips were thoroughly washed with PBS and mounted on glass slides with Mowiol mounting medium. The slides were then imaged on a FluoView FV1000 laser scanning microscope (Olympus) with 60x UPLSAPO 1.35NA (Olympus) oil immersion objective.

### Surface plasmon resonance

The SPR experiments were performed using a Biacore T200 system (GE Healthcare), and Series S CM5 or SA chips with 4 flow cells each.

#### Vinculin-binding experiment

Lyophilized vinculin was dissolved in flow buffer to a concentration of 1 mg/ml and covalently coupled onto the carboxymethylated dextran matrix of CM5 sensor chips using standard amine coupling. The flow buffer contained 10 mM HEPES, 150 mM NaCl, 3 mM EDTA, 0.05% surfactant P20. The chip was activated with an injection of 70 μl of a mixture of 0.05 M EDC and 0.2 M NHS, followed by the injection of approximately 70 μl of 10 μg/ml vinculin in 10 mM sodium acetate, pH 5.0 in one flow cell to yield a response of 7200 RU. Remaining reactive groups in the channel were blocked with an injection of 100 μl ethanolamine. Binding experiments were performed by injecting peptides for 120 s at a flow rate of 20 μl/min, at a concentration of 1 mM in the flow buffer over the chip surface to monitor association kinetics, followed by buffer flow for 120 s to measure dissociation kinetics. Between experiments, the surface was regenerated by injecting a 60 second pulse of 10 mM glycine pH 2.

#### Syntenin-binding experiment

Using the inverse of the approach from the previous experiment, 4 biotinylated peptides were immobilised on individual streptavidin surfaces of a Biacore SA chip. Each peptide was injected in flow buffer for 420 s at a flow rate of 10μl/min, at a concentration of 1 μM. Total immobilisation for each peptide was 500–600 RU. Syntenin-1 was then injected at a concentration of 31.25 nM in flow buffer. Chip surface regeneration was attempted by injecting a 60 second pulse of 0.25% SDS/20 mM EDTA, but was unsuccessful.

### Statistical analysis

All statistical analysis was performed using R, python, and Rpy2. All statistical tests of significance used a one-tailed or two-tailed Wilcoxon signed-rank test compared with controls. We chose the Wilcoxon signed-rank test because of the non-normal distribution of data points. We used a one-tailed test in each analysis, because we were testing for inhibition of platelet activation in one set of experiments, and for activation of resting platelets in another set. Where the number of replicates is large enough a two tailed test is used. Error bars represent the standard error of the mean.

## Supporting information

S1 Fig Aggregation results before and after TRAP activation for all peptides. (PDF)

S2 Fig Sequence-specificity of the syndecan-4 derived peptide pal-SDC4_tail. (PDF)

S3 Fig Physical interaction of syntenin-1 with peptide from the tail of syndecan 4. (PDF)

S4 Fig Phosphorylation changes during platelet activation in response to peptide. (PDF)

S5 Fig Chimeric peptides from syndecan 4 cytoplasmic region. (PDF)

S6 Fig Effects of combining pal-ITGA2B_JM and tat peptides in tests for synergy. (PDF)

S7 Fig Effects of combinations of peptides on platelet activation. (PDF)

S8 Fig Effects of combinations of peptides on inhibition of platelet activation. (PDF)

S9 Fig Intracellular localisation of tat-ACTN1-VBS peptide. (PDF)

S10 Fig Differential platelet activation by ACTN1_VBS peptide depending on N or C terminal addition of the tat cell-penetrating peptide. (PDF)

S1 Table Phenotypic consequences of deleting adhesome components. (PDF)

S2 Table Peptide activities & literature-described interactions of syndecan peptide regions with protein interaction partners. (PDF)

S3 Table Effects of chimeric peptides between integrin alpha and other adhesome components. (PDF)

S4 Table Inhibitory effects of peptide combinations on the activation of platelets: Comparison with peptides at single concentrations. (PDF)

S5 Table Inhibitory effects of peptide combinations on TRAP activation of platelets: Comparison with peptides at double concentrations. (PDF)

S1 Data Excel-formatted workbook of adhesome of datasets used in generating figures. (XLSX)

## References

[1] Van Roey K, Gibson TJ, Davey NE. Motif switches: decision-making in cell regulation. Curr Opin Struct Biol. 2012;22: 378–85. 10.1016/j.sbi.2012.03.004 22480932

[2] Zaidel-Bar R, Geiger B. The switchable integrin adhesome. J Cell Sci. 2010;123: 1385–8. 10.1242/jcs.066183 20410370 PMC2858016

[3] Raab M, Daxecker H, Edwards RJ, Treumann A, Murphy D, Moran N. Protein interactions with the platelet integrin αIIb regulatory motif. Proteomics. 2010;10: 2790–800. 10.1002/pmic.200900621 20486118

[4] Inomata M, Hayashi M, Ohno-Iwashita Y, Tsubuki S, Saido TC, Kawashima S. Involvement of calpain in integrin-mediated signal transduction. Arch Biochem Biophys. 1996;328: 129–34. 10.1006/abbi.1996.0152 8638921

[5] Liu S, Calderwood DA, Ginsberg MH. Integrin Cytoplasmic Domain-Binding Proteins. J Cell Sci. 2000;113: 3563–3571. 11017872 10.1242/jcs.113.20.3563

[6] Anthis NJ, Campbell ID. The tail of integrin activation. Trends Biochem Sci. 2011;36: 191–8. 10.1016/j.tibs.2010.11.002 21216149 PMC3078336

[7] Geiger T, Zaidel-Bar R. Opening the floodgates: proteomics and the integrin adhesome. Curr Opin Cell Biol. 2012;24: 562–8. 10.1016/j.ceb.2012.05.004 22728062

[8] Zaidel-Bar R, Itzkovitz S, Ma’ayan A, Iyengar R, Geiger B. Functional atlas of the integrin adhesome. Nat Cell Biol. Nature Publishing Group; 2007;9: 858–67. 10.1038/ncb0807-858 17671451 PMC2735470

[9] Shattil SJ, Kim C, Ginsberg MH. The final steps of integrin activation: the end game. Nat Rev Mol Cell Biol. 2010;11: 288–300. 10.1038/nrm2871 20308986 PMC3929966

[10] Coller BS. Blockade of platelet GPIIb/IIIa receptors as an antithrombotic strategy. Circulation. 1995;92: 2373–80. 7586333 10.1161/01.cir.92.9.2373

[11] Schwab N, Schneider-Hohendorf T, Wiendl H. Therapeutic uses of anti-α4-integrin (anti-VLA-4) antibodies in multiple sclerosis. Int Immunol. 2015;27: 47–53. 10.1093/intimm/dxu096 25326459

[12] Shattil SJ, Newman PJ. Integrins: dynamic scaffolds for adhesion and signaling in platelets. Blood. 2004;104: 1606–15. 10.1182/blood-2004-04-1257 15205259

[13] Cox D, Brennan M, Moran N. Integrins as therapeutic targets: lessons and opportunities. Nat Rev Drug Discov. Nature Publishing Group, a division of Macmillan Publishers Limited. All Rights Reserved.; 2010;9: 804–20. 10.1038/nrd3266 20885411

[14] Nanda SY, Hoang T, Patel P, Zhang H. Vinculin regulates assembly of talin: β3 integrin complexes. J Cell Biochem. 2014;115: 1206–16. 10.1002/jcb.24772 24446374

[15] Craig R, Cortens JP, Beavis RC. Open source system for analyzing, validating, and storing protein identification data. J Proteome Res. 2004;3: 1234–42. 10.1021/pr049882h 15595733

[16] Boyanova D, Nilla S, Birschmann I, Dandekar T, Dittrich M. PlateletWeb: a systems biologic analysis of signaling networks in human platelets. Blood. 2012;119: e22–e34. 10.1182/blood-2011-10-387308 22123846

[17] Davey NE, Van Roey K, Weatheritt RJ, Toedt G, Uyar B, Altenberg B, et al Attributes of short linear motifs. Mol Biosyst. Royal Society of Chemistry; 2012;8: 268–81. 10.1039/c1mb05231d 21909575

[18] Mooney C, Pollastri G, Shields DC, Haslam NJ. Prediction of short linear protein binding regions. J Mol Biol. 2012;415: 193–204. 10.1016/j.jmb.2011.10.025 22079048

[19] Davey NE, Cowan JL, Shields DC, Gibson TJ, Coldwell MJ, Edwards RJ. SLiMPrints: conservation-based discovery of functional motif fingerprints in intrinsically disordered protein regions. Nucleic Acids Res. 2012;40: 10628–41. 10.1093/nar/gks854 22977176 PMC3510515

[20] O’Callaghan K, Kuliopulos A, Covic L. Turning receptors on and off with intracellular pepducins: new insights into G-protein-coupled receptor drug development. J Biol Chem. 2012;287: 12787–96. 10.1074/jbc.R112.355461 22374997 PMC3339939

[21] Stephens G. A Sequence within the Cytoplasmic Tail of GpIIb Independently Activates Platelet Aggregation and Thromboxane Synthesis. J Biol Chem. 1998;273: 20317–20322. 10.1074/jbc.273.32.20317 9685382

[22] Aylward K, Meade G, Ahrens I, Devocelle M, Moran N. A novel functional role for the highly conserved alpha-subunit KVGFFKR motif distinct from integrin alphaIIbbeta3 activation processes. J Thromb Haemost. 2006;4: 1804–12. 10.1111/j.1538-7836.2006.02041.x 16879224

[23] Koloka V, Christofidou ED, Vaxevanelis S, Dimitriou AA, Tsikaris V, Tselepis AD, et al A palmitoylated peptide, derived from the acidic carboxyl-terminal segment of the integrin alphaIIb cytoplasmic domain, inhibits platelet activation. Platelets. Informa UK Ltd UK; 2008;19: 502–11. 10.1080/09537100802266875 18979362

[24] Dimitriou AA, Stathopoulos P, Mitsios J V., Sakarellos-Daitsiotis M, Goudevenos J, Tsikaris V, et al Inhibition of platelet activation by peptide analogs of the β3-intracellular domain of platelet integrin αIIbβ3 conjugated to the cell-penetrating peptide Tat(48–60), Platelets, Informa Healthcare [Internet]. Informa UK Ltd UK; 23 11 2009. 10.3109/09537100903324219 19863457

[25] Legate KR, Fässler R. Mechanisms That Regulate Adaptor Binding to Β-Integrin Cytoplasmic Tails. J Cell Sci. 2009;122: 187–198. 10.1242/jcs.041624 19118211

[26] Otey CA, Vasquez GB, Burridge K, Erickson BW. Mapping of the Alpha-Actinin Binding Site Within the Beta 1 Integrin Cytoplasmic Domain. J Biol Chem. 1993;268: 21193–21197. 7691808

[27] Morgan MR, Humphries MJ, Bass MD. Synergistic control of cell adhesion by integrins and syndecans. Nat Rev Mol Cell Biol. 2007;8: 957–69. 10.1038/nrm2289 17971838 PMC3329926

[28] Sheibani N, Tang Y, Sorenson CM. Paxillin’s LD4 motif interacts with bcl-2. J Cell Physiol. 2008;214: 655–61. 10.1002/jcp.21256 17786945

[29] Bois PRJ, Borgon RA, Vonrhein C, Izard T. Structural dynamics of alpha-actinin-vinculin interactions. Mol Cell Biol. 2005;25: 6112–22. 10.1128/MCB.25.14.6112-6122.2005 15988023 PMC1168820

[30] Brindle NP, Holt MR, Davies JE, Price CJ, Critchley DR. The focal-adhesion vasodilator-stimulated phosphoprotein (VASP) binds to the proline-rich domain in vinculin. Biochem J. 1996;318 (Pt 3: 753–7. 8836115 10.1042/bj3180753 PMC1217682

[31] Walders-Harbeck B. The vasodilator-stimulated phosphoprotein promotes actin polymerisation through direct binding to monomeric actin. FEBS Lett. 2002;529: 275–280. 10.1016/S0014-5793(02)03356-2 12372613

[32] Wu H-Y, Tomizawa K, Matsushita M, Lu Y-F, Li S-T, Matsui H. Poly-arginine-fused calpastatin peptide, a living cell membrane-permeable and specific inhibitor for calpain. Neurosci Res. 2003;47: 131–135. 10.1016/S0168-0102(03)00195-0 12941454

[33] Tompa P, Mucsi Z, Orosz G, Friedrich P. Calpastatin subdomains A and C are activators of calpain. J Biol Chem. 2002;277: 9022–6. 10.1074/jbc.C100700200 11809743

[34] Pearson MA, Reczek D, Bretscher A, Karplus PA. Structure of the ERM Protein Moesin Reveals the FERM Domain Fold Masked by an Extended Actin Binding Tail Domain. Cell. 2000;101: 259–270. 10.1016/S0092-8674(00)80836-3 10847681

[35] Terawaki S, Maesaki R, Hakoshima T. Structural basis for NHERF recognition by ERM proteins. Structure. 2006;14: 777–89. 10.1016/j.str.2006.01.015 16615918

[36] Thomas JW. SH2- and SH3-mediated Interactions between Focal Adhesion Kinase and Src. J Biol Chem. 1998;273: 577–583. 10.1074/jbc.273.1.577 9417118

[37] Hashimoto S, Hirose M, Hashimoto A, Morishige M, Yamada A, Hosaka H, et al Targeting AMAP1 and cortactin binding bearing an atypical src homology 3/proline interface for prevention of breast cancer invasion and metastasis. Proc Natl Acad Sci U S A. 2006;103: 7036–41. 10.1073/pnas.0509166103 16636290 PMC1459014

[38] Landesberg R, Burke A, Pinsky D, Katz R, Vo J, Eisig SB, et al Activation of platelet-rich plasma using thrombin receptor agonist peptide. J Oral Maxillofac Surg. 2005;63: 529–35. 10.1016/j.joms.2004.12.007 15789326

[39] Baciu PC, Saoncella S, Lee SH, Denhez F, Leuthardt D, Goetinck PF. Syndesmos, a protein that interacts with the cytoplasmic domain of syndecan-4, mediates cell spreading and actin cytoskeletal organization. J Cell Sci. 2000;113 Pt 2: 315–24. 10633082 10.1242/jcs.113.2.315

[40] Woods A. Syndecan-4 Proteoglycan Regulates the Distribution and Activity of Protein Kinase C. J Biol Chem. 1997;272: 8133–8136. 10.1074/jbc.272.13.8133 9079625

[41] Grootjans JJ, Zimmermann P, Reekmans G, Smets A, Degeest G, Dürr J, et al Syntenin, a PDZ protein that binds syndecan cytoplasmic domains. Proc Natl Acad Sci U S A. 1997;94: 13683–8. 9391086 10.1073/pnas.94.25.13683 PMC28366

[42] Lombardi F, Golla K, Fitzpatrick DJ, Casey FP, Moran N, Shields DC. Discovering anti-platelet drug combinations with an integrated model of activator-inhibitor relationships, activator-activator synergies and inhibitor-inhibitor synergies. PLoS Comput Biol. 2015;11: e1004119 10.1371/journal.pcbi.1004119 25875950 PMC4405222

[43] Berenbaum MC. What is synergy? Pharmacol Rev. 1989;41: 93–141. 2692037

[44] Johannessen L, Remsberg J, Gaponenko V, Adams KM, Barchi JJ, Tarasov SG, et al Peptide Structure Stabilization by Membrane Anchoring and its General Applicability to the Development of Potent Cell-Permeable Inhibitors. ChemBioChem. Wiley-Blackwell; 2011;12: 914–921. 10.1002/cbic.201000563 21365731 PMC3493468

[45] Dosztanyi Z, Meszaros B, Simon I. ANCHOR: web server for predicting protein binding regions in disordered proteins. Bioinformatics. 2009;25: 2745–2746. 10.1093/bioinformatics/btp518 19717576 PMC2759549

[46] Edwards RJ, Davey NE, Shields DC. SLiMFinder: a probabilistic method for identifying over-represented, convergently evolved, short linear motifs in proteins. PLoS One. Public Library of Science; 2007;2: e967 10.1371/journal.pone.0000967 17912346 PMC1989135

[47] Neduva V, Russell RB. DILIMOT: discovery of linear motifs in proteins. Nucleic Acids Res. 2006;34: W350–W355. 10.1093/nar/gkl159 16845024 PMC1538856

[48] Gouw M, Michael S, Sámano-Sánchez H, Kumar M, Zeke A, Lang B, et al The eukaryotic linear motif resource– 2018 update. Nucleic Acids Res. 2018;46: D428–D434. 10.1093/nar/gkx1077 29136216 PMC5753338

[49] Mirabello C, Pollastri G. Porter, PaleAle 4.0: high-accuracy prediction of protein secondary structure and relative solvent accessibility. Bioinformatics. 2013;29: 2056–2058. 10.1093/bioinformatics/btt344 23772049

[50] Drozdetskiy A, Cole C, Procter J, Barton GJ. JPred4: a protein secondary structure prediction server. Nucleic Acids Res. Oxford University Press; 2015;43: W389–W394. 10.1093/nar/gkv332 25883141 PMC4489285

[51] Jiang Q, Jin X, Lee S-J, Yao S. Protein secondary structure prediction: A survey of the state of the art. J Mol Graph Model. Elsevier; 2017;76: 379–402. 10.1016/j.jmgm.2017.07.015 28763690

[52] Dosztányi Z. Prediction of protein disorder based on IUPred. Protein Sci. 2018;27: 331–340. 10.1002/pro.3334 29076577 PMC5734386

[53] Wagner CL, Mascelli MA, Neblock DS, Weisman HF, Coller BS, Jordan RE. Analysis of GPIIb/IIIa receptor number by quantification of 7E3 binding to human platelets. Blood. 1996;88: 907–14. 8704248

[54] Haslam NJ, Shields DC. Peptide-binding domains: are limp handshakes safest? Sci Signal. 2012;5: pe40 10.1126/scisignal.2003372 23012652

[55] Nelson ES, Folkmann AW, Henry MD, DeMali KA. Vinculin activators target integrins from within the cell to increase melanoma sensitivity to chemotherapy. Mol Cancer Res. 2011;9: 712–23. 10.1158/1541-7786.MCR-10-0599 21460181 PMC3134390

[56] Horton ER, Humphries JD, James J, Jones MC, Askari JA, Humphries MJ. The integrin adhesome network at a glance. J Cell Sci. 2016;129: 4159–4163. 10.1242/jcs.192054 27799358 PMC5117201

