# Supplementary material for: Computational and experimental analysis of bioactive peptide linear motifs in the integrin adhesome
Source: PLoS One. 2019 Jan 28;14(1):e0210337. doi: 10.1371/journal.pone.0210337 (PMC6349357; doi:10.1371/journal.pone.0210337)
Supplement: S1 Fig — (PDF) [file pone.0210337.s001.pdf]

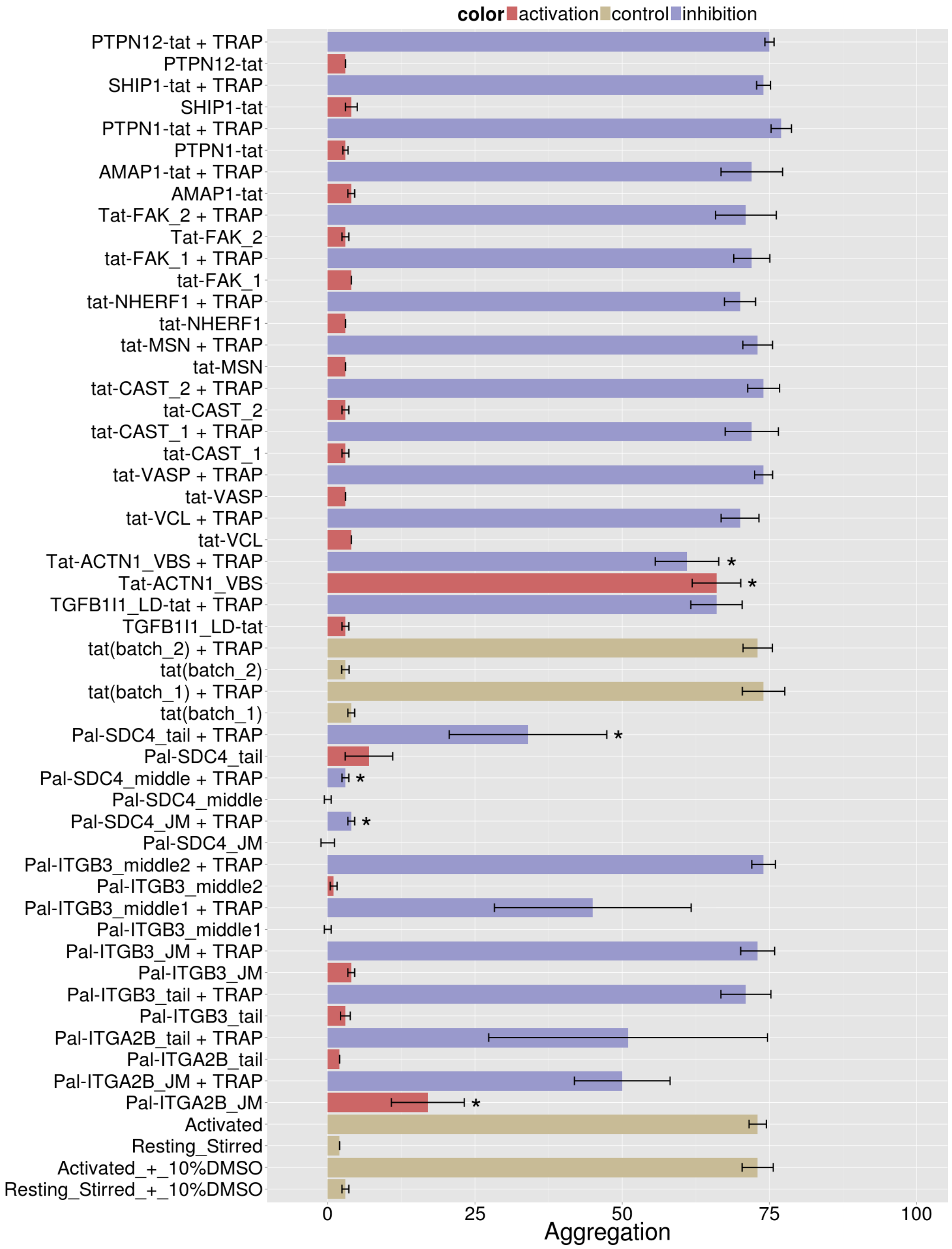

*: P<=0.05, one tailed Wilcoxon test compared with the resting stirred, and TRAP activated for pal peptides or tat and tat + TRAP activated for tat peptides.

S1 Fig Aggregation results before and after TRAP activation for all peptides. Palmitylated (pal) peptide=20µM, tat peptide=50µM, TRAP: 4µM. Error bars: standard error of the mean.
