# Supplementary material for: Computational and experimental analysis of bioactive peptide linear motifs in the integrin adhesome
Source: PLoS One. 2019 Jan 28;14(1):e0210337. doi: 10.1371/journal.pone.0210337 (PMC6349357; doi:10.1371/journal.pone.0210337)
Supplement: S2 Fig — (PDF) [file pone.0210337.s002.pdf]

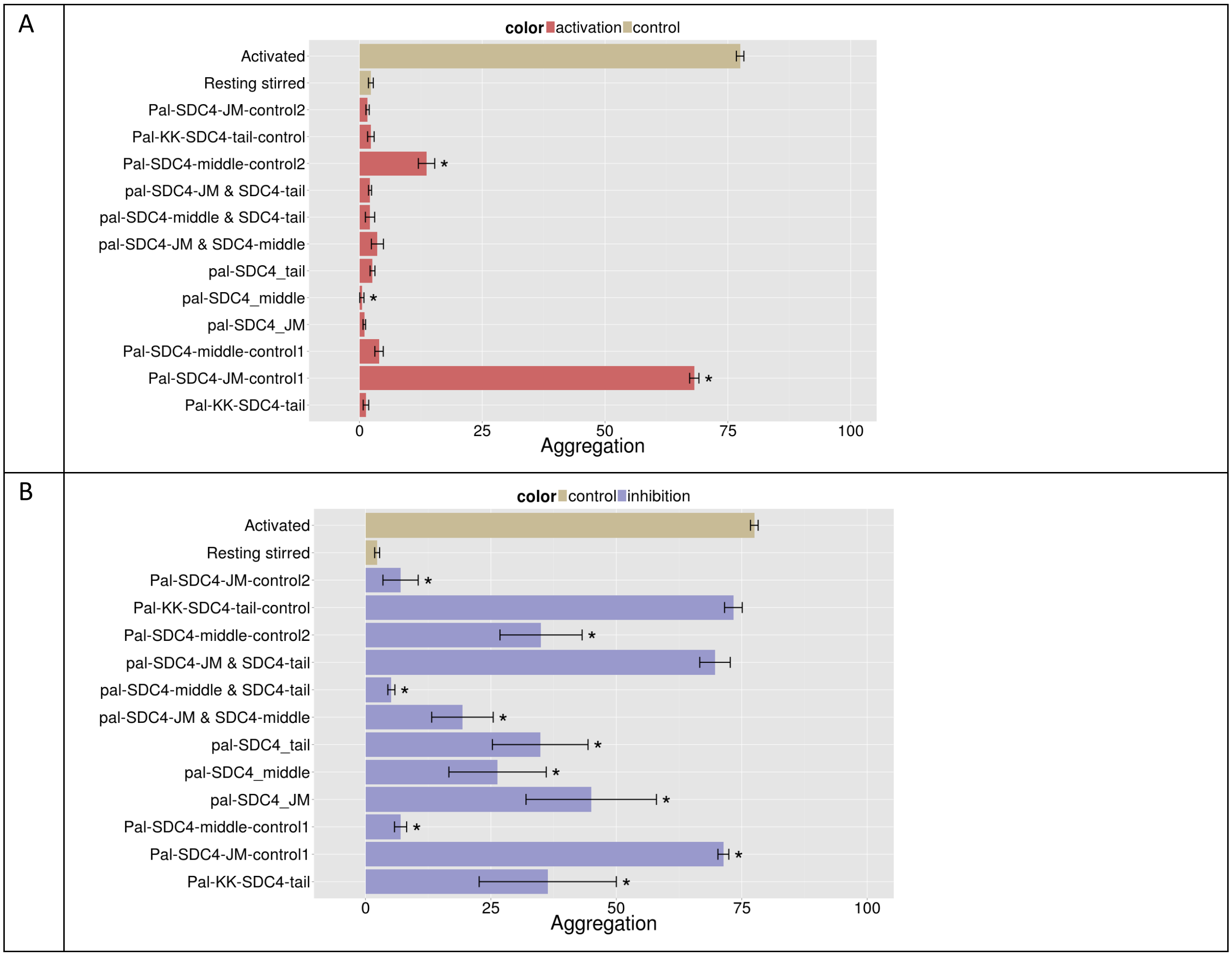

S2 Fig. Sequence-specificity of the syndecan-4 derived peptide pal-SDC4_tail. Peptide effects (n=6 donors, * P <=0.05, Two tailed Wilcoxon signed-rank test compared with Activated or Resting Stirred) compared with scrambled controls (n=3 donors, 1-tailed test) (A) Effects on activation of aggregation, observations at 20µM. No strong effects were seen at 1 and 5 µM except for the activatory effect of pal-SDC4_JM_Control1 which showed activatory effects at all three doses. (B) effects on inhibition of platelet aggregation induced by 4 µM TRAP.
