# Supplementary material for: Computational and experimental analysis of bioactive peptide linear motifs in the integrin adhesome
Source: PLoS One. 2019 Jan 28;14(1):e0210337. doi: 10.1371/journal.pone.0210337 (PMC6349357; doi:10.1371/journal.pone.0210337)
Supplement: S3 Fig — (PDF) [file pone.0210337.s003.pdf]

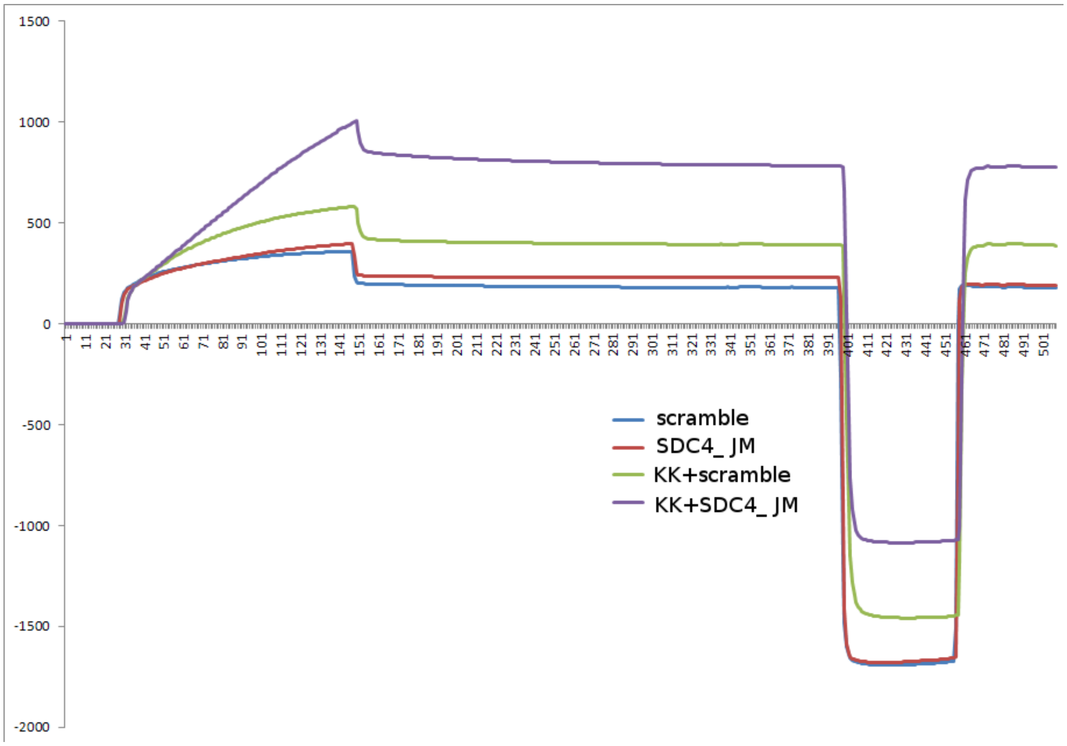

S3 Fig: Physical interaction of Syntenin-1 with peptide from the tail of Syndecan 4. Surface plasmon resonance sensorgrams for protein injection at 31.24nM onto immobilised peptide on a flow cell of a CM5 sensor chip (Original data unsubtracted from control). The N-terminus of each peptide was bound to the SPR surface, mimicking the likely orientation of pal-peptides, where the palmityl group is embedded in the membrane and the peptide is free to interact with membrane-adjacent proteins. Syntenin-1 bound to all peptides, including scrambled, at low concentration (31.25nM) in a high affinity interaction. We were unable to report KD values because scrambled peptides bound tightly and further cycles were prevented due to poor regeneration of the sensorgram. The relative binding affinity indicates a priority of binding of KK+SDC4_JM > SDC4_JM > scrambled peptides.
