# Supplementary material for: Computational and experimental analysis of bioactive peptide linear motifs in the integrin adhesome
Source: PLoS One. 2019 Jan 28;14(1):e0210337. doi: 10.1371/journal.pone.0210337 (PMC6349357; doi:10.1371/journal.pone.0210337)
Supplement: S4 Fig — (PDF) [file pone.0210337.s004.pdf]

A

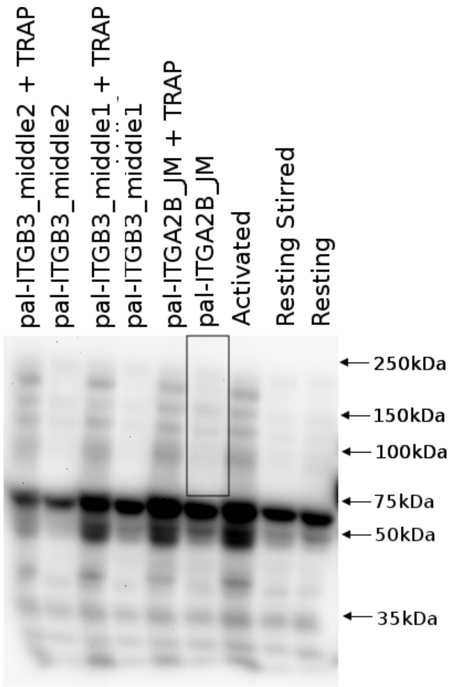

B

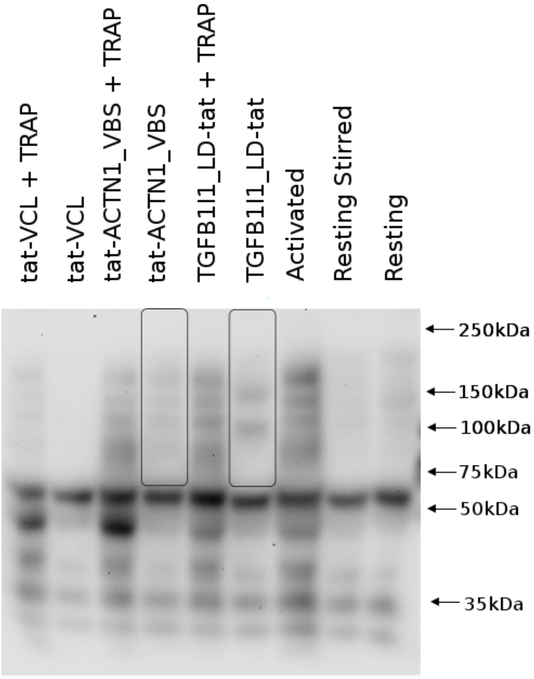

C

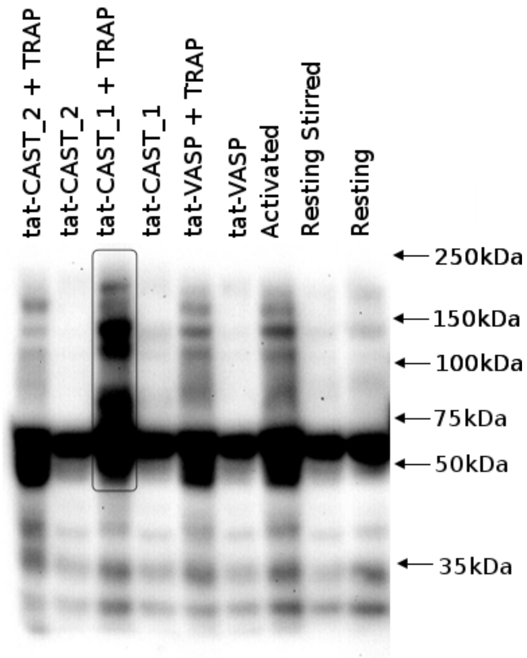

D

Replicate	1	2	3	Average
tat-CAST1 + TRAP density	80	58.7	54.9	64.53
Activated density	19.4	41.2	45	26.4
relative density	4.12	1.42	1.22	2.44
p-value				0.029

S4 Fig: Phosphorylation changes during platelet activation in response to peptide. Phosphotyrosine western blots (4G10 antibody) of resting platelets with added peptide, and after TRAP stimulation of platelets pre-incubated with peptide. Palmitylated (pal) peptides:20µM; tat peptides:50µM; TRAP: 4µM. Results are shown where at least one peptide is associated with a change in phosphorylation profile (boxed) for (A) pal-ITGA2B_JM peptide (B) tat-ACTN1_VBS and TGFB1I1_LD-tat peptides (C) tat-CAST-1 peptide. (D) shows the relative density of the tat-CAST-1 peptide lane to the activated control sample.
