# Supplementary material for: Computational and experimental analysis of bioactive peptide linear motifs in the integrin adhesome
Source: PLoS One. 2019 Jan 28;14(1):e0210337. doi: 10.1371/journal.pone.0210337 (PMC6349357; doi:10.1371/journal.pone.0210337)
Supplement: S5 Fig — (PDF) [file pone.0210337.s005.pdf]

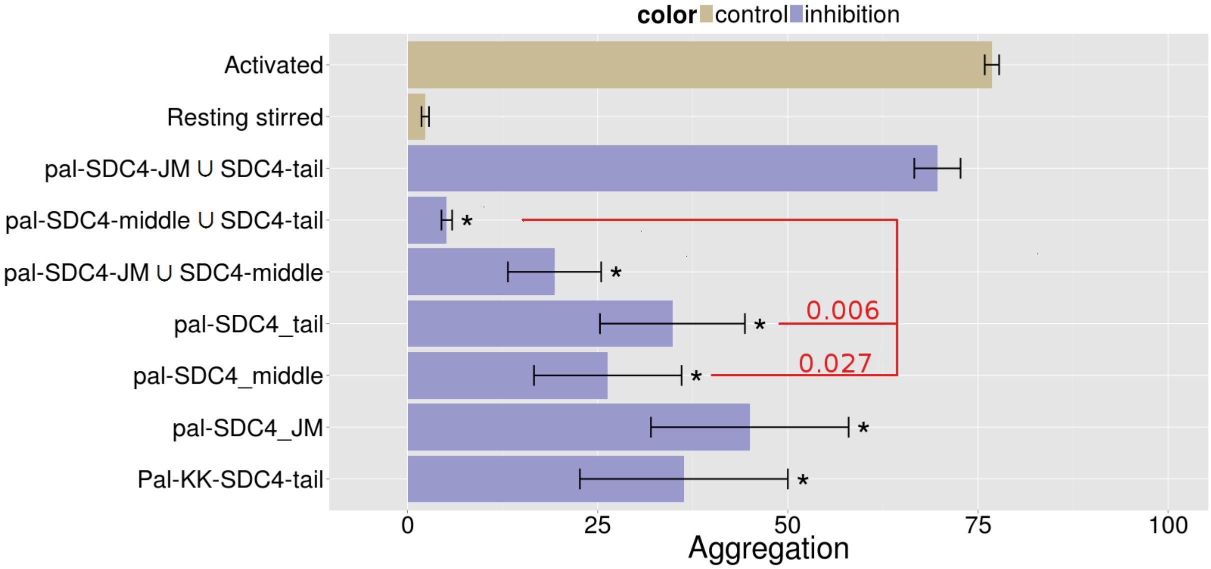

S5 Fig: Chimeric peptides from Syndecan 4 cytoplasmic region. Inhibition of platelet activation in response to TRAP (peptide:20μM,TRAP=4μM, n=6):* $P \leq 0.05$, two-tailed Wilcoxon signed-rank test compared with TRAP activated control, red: p-value from comparisons of indicated groups. Error bars: standard error.
