# Supplementary material for: Computational and experimental analysis of bioactive peptide linear motifs in the integrin adhesome
Source: PLoS One. 2019 Jan 28;14(1):e0210337. doi: 10.1371/journal.pone.0210337 (PMC6349357; doi:10.1371/journal.pone.0210337)
Supplement: S6 Fig — (PDF) [file pone.0210337.s006.pdf]

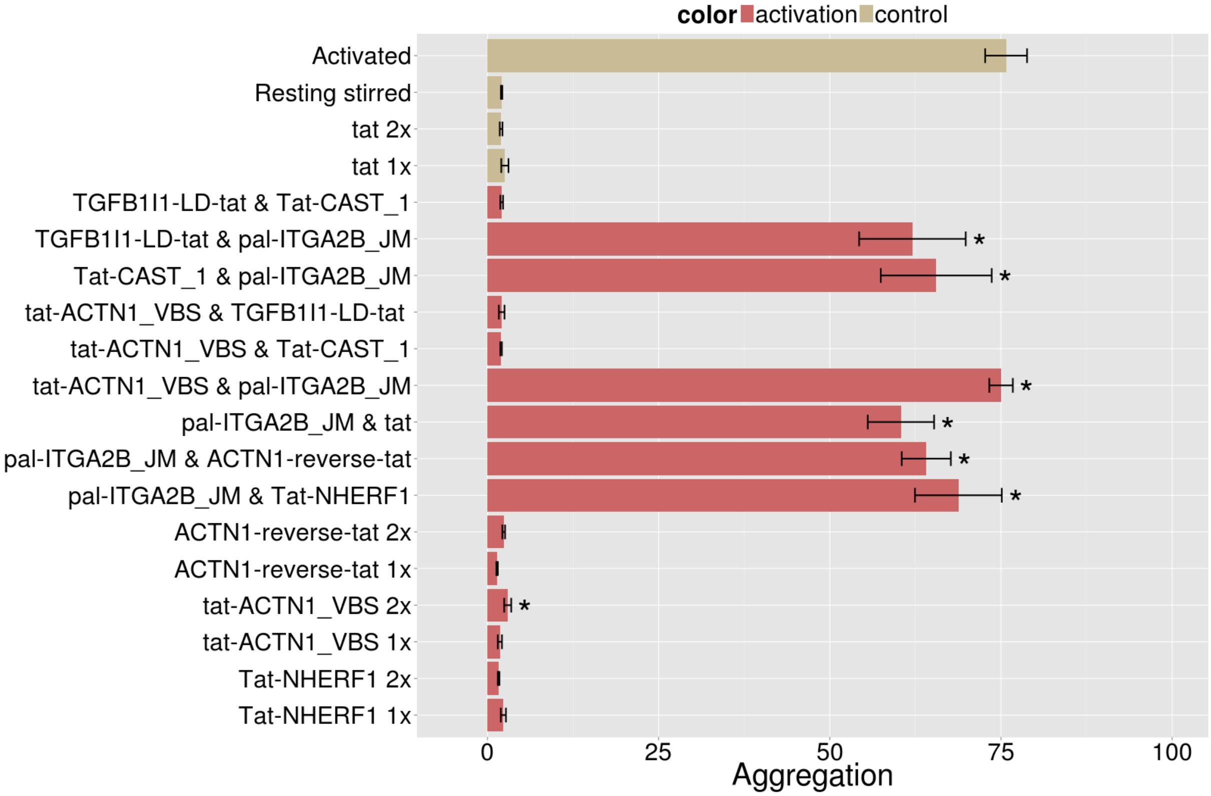

S6 Fig: Effects of combining pal-ITGA2B_JM and tat peptides in tests for synergy. Palmitylated **(**Pal) peptide concentrations are 10µM; tat peptide concentrations are 20µM; TRAP Control: 2µM; n=3. Error bars indicate the standard error of the mean. * P <=0.05, One tailed Wilcoxon signed-rank test compared with the tat 2x + low dose TRAP. The strong effect of tat alone suggests that the strong synergism of the other tat containing peptides reflects a synergy of the tat peptide region alone.
