# Supplementary material for: Computational and experimental analysis of bioactive peptide linear motifs in the integrin adhesome
Source: PLoS One. 2019 Jan 28;14(1):e0210337. doi: 10.1371/journal.pone.0210337 (PMC6349357; doi:10.1371/journal.pone.0210337)
Supplement: S7 Fig — (PDF) [file pone.0210337.s007.pdf]

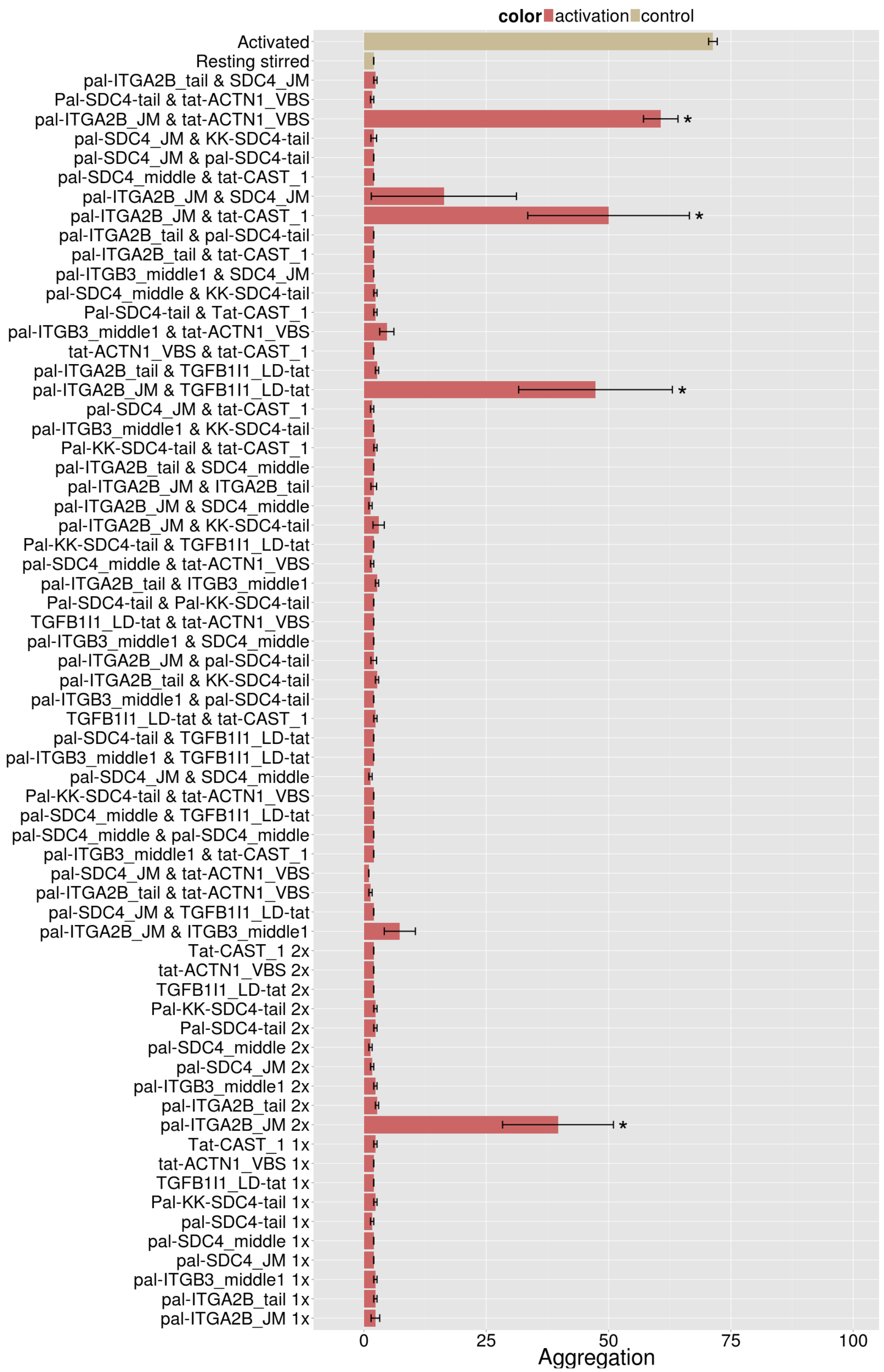

S7 Fig: Effects of combinations of peptides on platelet activation. Platelet aggregation induced in resting platelets after 6 minutes incubation with peptides, quantified as optical density using an aggregometer, n=3. *: P<=0.05, one tailed Wilcoxon test compared with Resting Stirred peptide.
