# Supplementary material for: Computational and experimental analysis of bioactive peptide linear motifs in the integrin adhesome
Source: PLoS One. 2019 Jan 28;14(1):e0210337. doi: 10.1371/journal.pone.0210337 (PMC6349357; doi:10.1371/journal.pone.0210337)
Supplement: S8 Fig — (PDF) [file pone.0210337.s008.pdf]

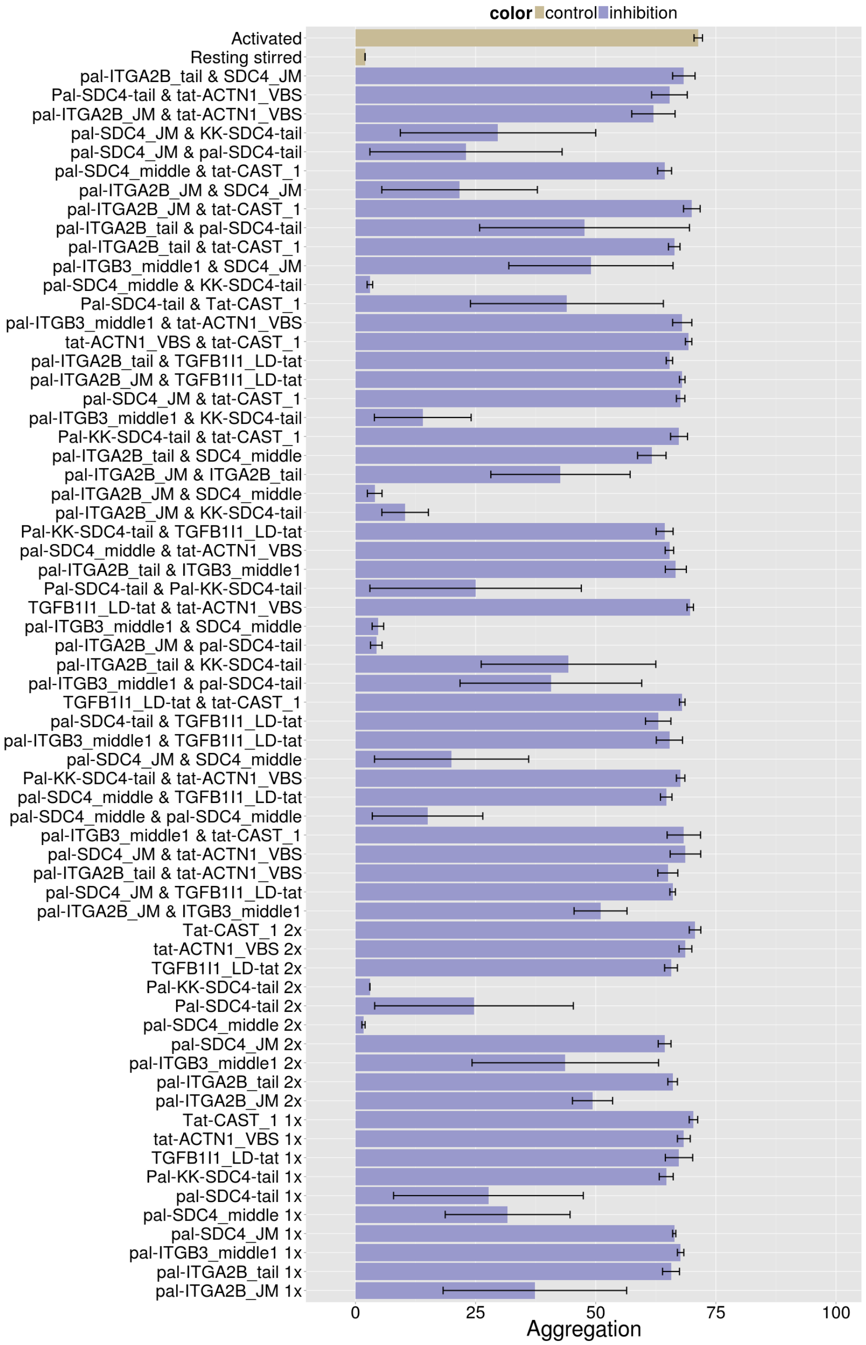

S8 Fig: Effects of combinations of peptides on inhibition of platelet activation. inhibition of TRAP (2 µM) activation of platelets after pre-incubation of the platelets for 6 minutes with the peptides, quantified as optical density using an aggregometer, n=3
