# Supplementary material for: Computational and experimental analysis of bioactive peptide linear motifs in the integrin adhesome
Source: PLoS One. 2019 Jan 28;14(1):e0210337. doi: 10.1371/journal.pone.0210337 (PMC6349357; doi:10.1371/journal.pone.0210337)
Supplement: S9 Fig — (PDF) [file pone.0210337.s009.pdf]

(A)

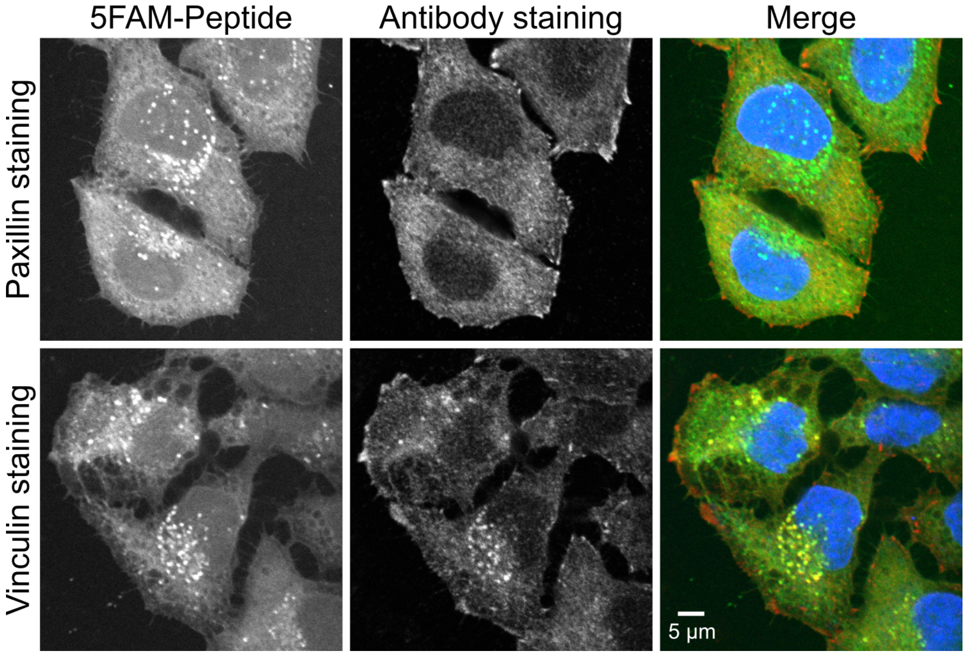

(B)

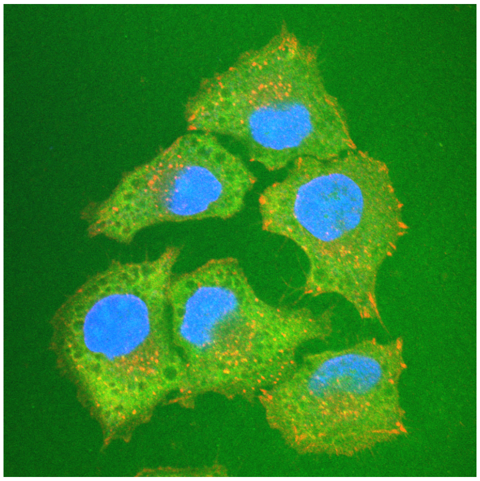

S9 Fig: Intracellular localisation of tat-ACTN1-VBS peptide

(A) HeLa cells were treated with 5FAM-labeled tat-ACTN1-VBS peptide and immuno-stained with either vinculin (bottom) or paxillin (top) antibody. Blue color represents nuclear stain Hoechs33342; green color represents tat-ACTN1-VBS peptide, conjugated with the 5FAM fluorophore at the N terminus; red color represents paxillin or vinculin staining, as labeled; yellow indicates co-localization of protein and peptide.

(B) As above, except green represents 5FAM-labeled tat control peptide, and red represents vinculin. This shows a similar distribution for vinculin as in (A), but the 5FAM-tat is localizing throughout the cell, and not co-localizing with vinculin.
