# Supplementary material for: Computational and experimental analysis of bioactive peptide linear motifs in the integrin adhesome
Source: PLoS One. 2019 Jan 28;14(1):e0210337. doi: 10.1371/journal.pone.0210337 (PMC6349357; doi:10.1371/journal.pone.0210337)

S10 Fig: Differential platelet activation by ACTN1_VBS peptide depending on N or C terminal addition of the tat cell-penetrating peptide. Activation screening of the reverse tat (peptide:50µM. Activated control: platelet stimulated by 4µM TRAP):* P $\leq$ 0.05, One tailed Wilcoxon signed-rank test compared with the tat control. Error bars indicate the standard error of the mean. n=4.

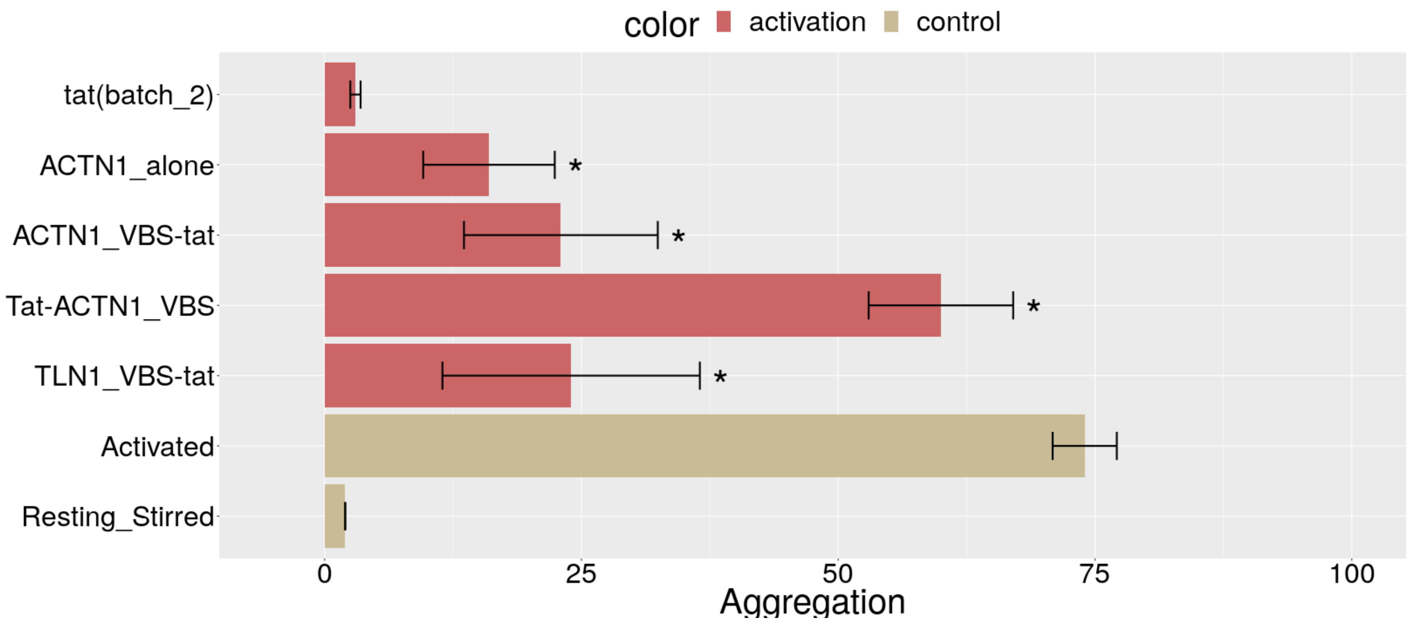

Supplement: S10 Fig — (PDF) [file pone.0210337.s010.pdf]
