# Supplementary material for: Computational and experimental analysis of bioactive peptide linear motifs in the integrin adhesome
Source: PLoS One. 2019 Jan 28;14(1):e0210337. doi: 10.1371/journal.pone.0210337 (PMC6349357; doi:10.1371/journal.pone.0210337)
Supplement: S1 Table — (PDF) [file pone.0210337.s011.pdf]

| **Human gene name** | **Phenotype of double knockout of mouse orthologue**[1] |
|---|---|
| ITGA2B | bleeding platelets do not bind fibrinogen |
| ITGB3 | bleeding; reduced survival |
| SDC4 | delayed wound healing |
| TGFB1I1 | not known |
| ACTN1 | not known |
| VCL | embryonic lethal |
| VASP | hyperplasia of megakaryocytes |
| CAST | augmented DNA fragmentation |
| MSN | Normal |
| NHERF1 | Phosphate irregularities |
| FAK | Die during organogenesis |
| AMAP1 | Not known |
| PTPN1 | Lower adiposity, insulin sensitivity |
| INPP5D | Lack of transplant rejection |
| PTPN12 | Embryonic lethal |

**S1 Table. Phenotypic consequences of deleting adhesome components**
