# Supplementary material for: Computational and experimental analysis of bioactive peptide linear motifs in the integrin adhesome
Source: PLoS One. 2019 Jan 28;14(1):e0210337. doi: 10.1371/journal.pone.0210337 (PMC6349357; doi:10.1371/journal.pone.0210337)
Supplement: S2 Table — (PDF) [file pone.0210337.s012.pdf]

A	

Peptide	Aggregation	src	fyn	syndesmos	syntenin	PKC	PIP2
Pal-SDC4_JM	45	Y	Y	Y			
Pal-SDC4_middle	26			?		Y	Y
Pal-SDC4_tail	34				Y		
Pal-SDC4_JM$_{U}$SDC4_middle	19	Y	Y	Y		Y	Y
Pal-SDC4_middle$_{U}$SDC4_tail	5			?	Y	Y	Y
Pal-SDC4_JM$_{U}$SDC4_tail	69	Y	Y	Y	Y		

B	

Region	20μM	Sequence	Positive charges	Negative charges
pal-SDC4_JM	Inhibitor	RMKKKDEGSYD	4	3
pal-SDC4_JM Control 1	Activator (also at 1 and 5 μM)	YKDSMERDKGK	4	3
pal-SDC4_JM Control 2	Inhibitor	MGYRKEDKDKS	4	3
pal-SDC4_middle	Inhibitor	LGKKPIYKK	4	0
pal-SDC4_middle Control 1	Inhibitor and mild activator	KIKGPKLKY	4	0
pal-SDC4_middle Control 2	Inhibitor and mild activator	IGLPKKKYK	4	0
pal-SDC4_tail	Inhibitor	APTNEFYA	0	1
pal-KK-SDC4_tail	Inhibitor	KKAPTNEFYA	2	1
pal-KK-SDC4_tail Control 1	No effect	NTYAFKAKEP	2	1

S2 Table. Peptide activities & literature-described interactions of syndecan peptide regions with protein interaction partners.

(A) Inhibition effects of syndecan-derived peptides (Aggregation data is shown in more detail in Fig. S8). A subscripted U between two peptide region names denotes the chimerization of the two regions as a single peptide, and the indicated interactions are inferred from the component peptide regions. (B) Summary of effects of SDC4-derived palmitylated peptides and their scrambled controls.
