# Supplementary material for: Computational and experimental analysis of bioactive peptide linear motifs in the integrin adhesome
Source: PLoS One. 2019 Jan 28;14(1):e0210337. doi: 10.1371/journal.pone.0210337 (PMC6349357; doi:10.1371/journal.pone.0210337)
Supplement: S3 Table — (PDF) [file pone.0210337.s013.pdf]

Peptide Name	Sequence	Peptide Concentration			
		20µM		5µM	
		Activation	Inhibition	Activation	Inhibition
Pal-ITGA2b	pal-KVGFFKR	62*	69	66*	73
pal-ITGA2bJM2	pal-KVGAAKR	1	3*	1	28*
B3p ACTN1-VBS	pal-WEQLLTTIARTINEVENQI	1	73	2	76
B12p ITGA2b-JM ∪ ACTN1-VBS	pal-KVGFFKR-WEQLLTTIARTINEVENQI	7*	66	1	74
B13p ITGA2b-JM2 ∪ ACTN1-VBS	pal-KVGAAKR-WEQLLTTIARTINEVENQI	12	63*	3	75
A5p ITGB3-middle1	pal-FAKFEEERAR	2	68*	2	73
B15p ITGA2b-JM ∪ ITGB3-middle1	pal-KVGFFKR-FAKFEEERAR	4	71	2	72
B14p ITGA2b-JM2 ∪ ITGB3-middle1	pal-KVGAAKR-FAKFEEERAR	0	3*	2	71
A7p SDC4-JM	pal-RMKKKDEGSYD	1	73	2	75
B17p ITGA2b-JM2 ∪ SDC4-JM	pal-KVGFFKR-RMKKKDEGSYD	2	11*	1	66
B16p ITGA2b-JM ∪ SDC4-JM	pal-KVGAAKR-RMKKKDEGSYD	0	4*	3	72
A8p SDC4-middle	pal-LGKKPIYKK	1	41*	2	72
B18p ITGA2b-JM ∪ SDC4-middle	pal-KVGFFKR-LGKKPIYKK	13*	27*	61*	65
B19p ITGA2b-JM2 ∪ SDC4-middle	pal-KVGAAKR-LGKKPIYKK	3	6*	2	66*
B23p TGFB1I1-LD	pal-TLELDRLMASLS	3	70	2	73
B20p ITGA2b-JM ∪ TGFB1I1-LD	pal-KVGFFKR-TLELDRLMASLS	2	66*	2	71
B21p ITGA2b-JM2 ∪ TGFB1I1-LD	pal-KVGAAKR-TLELDRLMASLS	1	28*	2	70
Juxtamembrane (A)	Pal-RMKKKDEGSYD	1	45*	2	64
Variable (B)	Pal-LGKKPIYKK	1	26*	1	59

Tail (C)	Pal-APTNEFYA	2	34*	2	61
KK + Tail (C)	Pal-KKAPTNEFYA	1	36*	2	63
Juxtamembrane (A) ∪ Variable (B)	Pal-RMKKKDEGSYD-LGKKPIYKK	4	19*	2	61
Variable (B) ∪ Tail (C)	Pal-LGKKPIYKK-APTNEFYA	2	5*	3	63
Juxtamembrane (A) ∪ Tail (C)	Pal-RMKKKDEGSYD-APTNEFYA	2	69	2	63

S3 Table. Effects of chimeric peptides between integrin alpha and other adhesome components. Chimeras between two active peptides are indicated by their names separated by a "U". The "-" in their sequences indicates the point at which the two peptide sequences are joined. Significant aggregation and/or inhibition compared to control (resting platelets for activatory peptides; TRAP activated for inhibitory peptides) are indicated. n=3. *: $p<0.05$, one-tailed Wilcoxon. Blue highlights inhibition of platelet aggregation, red highlights platelet aggregation. Chimeras were also tested at 1µM and showed no significant effects. In addition to the activatory integrin peptide pal-ITGA2b-JM, we included in some chimeras the previously identified inhibitory integrin peptide obtained by substituting the two central phenylalanines of pal-ITGA2b-JM with alanines (see Raab M, Parthasarathi L, Treumann A, Moran N, Daxecker H. Differential binding of ICln in platelets to integrin-derived activating and inhibitory peptides. Biochem Biophys Res Commun. 2010;392: 258–63. doi:10.1016/j.bbrc.2009.12.088).
