# Supplementary material for: Computational and experimental analysis of bioactive peptide linear motifs in the integrin adhesome
Source: PLoS One. 2019 Jan 28;14(1):e0210337. doi: 10.1371/journal.pone.0210337 (PMC6349357; doi:10.1371/journal.pone.0210337)
Supplement: S4 Table — (PDF) [file pone.0210337.s014.pdf]

name	A 1x	B 1x	A + B (mix)	A vs mix pvalue	B Vs mix pvalue
pal-ITGA2B_tail & TGFB1I1_LD-tat	65.67	67.33	65.33	0.67	0.59
pal-ITGA2B_tail & pal-SDC4_middle	65.67	31.67	61.67	0.25	0.95
pal-ITGA2B_tail & pal-ITGB3_middle1	65.67	67.67	66.67	0.75	0.33
pal-ITGA2B_tail & pal-SDC4_JM	65.67	66.33	68.33	0.91	0.7
pal-ITGA2B_tail & tat-CAST_1	65.67	70.33	66.33	0.65	0.05*
pal-ITGA2B_tail & tat-ACTN1_VBS	65.67	68.33	65	0.41	0.19
pal-ITGA2B_tail & pal-KK-SDC4-tail	65.67	64.67	44.33	0.06	0.13
pal-ITGA2B_tail & pal-SDC4-tail	65.67	27.67	47.67	0.75	0.8
TGFB1I1_LD-tat & tat-CAST_1	67.33	70.33	68	0.8	0.06
TGFB1I1_LD-tat & tat-ACTN1_VBS	67.33	68.33	69.67	0.81	0.87
pal-SDC4_middle & pal-SDC4_middle	31.67	27.67	15	0.2	0.2
pal-SDC4_middle & TGFB1I1_LD-tat	31.67	67.33	64.67	1	0.25
pal-SDC4_middle & tat-CAST_1	31.67	70.33	64.33	1	0.05*
pal-SDC4_middle & tat-ACTN1_VBS	31.67	68.33	65.33	1	0.08
pal-SDC4_middle & pal-KK-SDC4-tail	31.67	64.67	3	0.05*	0.05*
pal-ITGB3_middle1 & TGFB1I1_LD-tat	67.67	67.33	65.33	0.41	0.5
pal-ITGB3_middle1 & pal-SDC4_middle	67.67	31.67	4.67	0.04*	0.05*
pal-ITGB3_middle1 & pal-SDC4_JM	67.67	66.33	49	0.25	0.33
pal-ITGB3_middle1 & tat-CAST_1	67.67	70.33	68.33	0.75	0.41
pal-ITGB3_middle1 & tat-ACTN1_VBS	67.67	68.33	68	0.82	0.5
pal-ITGB3_middle1 & pal-KK-SDC4-tail	67.67	64.67	14	0.04*	0.05*
pal-ITGB3_middle1 & pal-SDC4-tail	67.67	27.67	40.67	0.08	0.59
pal-SDC4_JM & TGFB1I1_LD-tat	66.33	67.33	66	0.41	0.75
pal-SDC4_JM & pal-SDC4_middle	66.33	31.67	20	0.04*	0.2
pal-SDC4_JM & tat-CAST_1	66.33	70.33	67.67	0.92	0.06
pal-SDC4_JM & tat-ACTN1_VBS	66.33	68.33	68.67	0.5	0.33
pal-SDC4_JM & pal-KK-SDC4-tail	66.33	64.67	29.67	0.33	0.35
pal-SDC4_JM & pal-SDC4-tail	66.33	27.67	23	0.04*	0.19
tat-ACTN1_VBS & tat-CAST_1	68.33	70.33	69.33	0.82	0.32
pal-ITGA2B_JM & pal-ITGA2B_tail	37.33	65.67	42.67	0.65	0.1
pal-ITGA2B_JM & TGFB1I1_LD-tat	37.33	67.33	68	0.87	0.8
pal-ITGA2B_JM & pal-SDC4_middle	37.33	31.67	4	0.13	0.05*
pal-ITGA2B_JM & pal-ITGB3_middle1	37.33	67.67	51	0.8	0.04*
pal-ITGA2B_JM & pal-SDC4_JM	37.33	66.33	21.67	0.5	0.04*
pal-ITGA2B_JM & tat-CAST_1	37.33	70.33	70	0.95	0.59
pal-ITGA2B_JM & tat-ACTN1_VBS	37.33	68.33	62	0.8	0.08
pal-ITGA2B_JM & pal-KK-SDC4-tail	37.33	64.67	10.33	0.35	0.05*
pal-ITGA2B_JM & pal-SDC4-tail	37.33	27.67	4.33	0.2	0.13
Pal-KK-SDC4-tail & tat-CAST_1	64.67	70.33	67.33	0.9	0.13

Pal-KK-SDC4-tail & tat-ACTN1_VBS	64.67	68.33	67.67	0.95	0.5
Pal-KK-SDC4-tail & TGFB1I1_LD-tat	64.67	67.33	64.33	0.5	0.41
pal-SDC4-tail & TGFB1I1_LD-tat	27.67	67.33	63	0.9	0.2
Pal-SDC4-tail & Tat-CAST_1	27.67	70.33	44	0.59	0.05*
Pal-SDC4-tail & tat-ACTN1_VBS	27.67	68.33	65.33	0.95	0.5
Pal-SDC4-tail & Pal-KK-SDC4-tail	27.67	64.67	25	0.33	0.33

S4 Table: Inhibitory effects of peptide combinations on the activation of platelets: comparison with peptides at single concentrations. A visual summary of this data is shown in Fig 3. Platelets were co-incubated with peptides (4 µM for pal-peptides; 10 µM for tat-peptides) individually or in combination, and then activated with TRAP (2 µM). * P<=0.05, two-tailed t-test: this is not a formal test of the probability of observing the association by chance, but is used here as a scale on which to highlight the most interesting effects.
