# Supplementary material for: Computational and experimental analysis of bioactive peptide linear motifs in the integrin adhesome
Source: PLoS One. 2019 Jan 28;14(1):e0210337. doi: 10.1371/journal.pone.0210337 (PMC6349357; doi:10.1371/journal.pone.0210337)
Supplement: S5 Table — (PDF) [file pone.0210337.s015.pdf]

name	A<br>2x	B<br>2x	A<br>+<br>B (mix)	A 2x<br>vs mix<br>p-value	B 2x<br>vs mix<br>p-value
pal-ITGA2B_tail & TGFB1I1_LD-tat	66	65.67	65.33	0.5	0.33
pal-ITGA2B_tail & pal-SDC4_middle	66	1.67	61.67	0.19	0.99
pal-ITGA2B_tail & pal-ITGB3_middle1	66	43.67	66.67	0.5	0.9
pal-ITGA2B_tail & pal-SDC4_JM	66	64.33	68.33	0.91	0.91
pal-ITGA2B_tail & tat-CAST_1	66	70.67	66.33	0.59	0.05*
pal-ITGA2B_tail & tat-ACTN1_VBS	66	68.67	65	0.33	0.09
pal-ITGA2B_tail & pal-KK-SDC4-tail	66	3	44.33	0.04*	0.99
pal-ITGA2B_tail & pal-SDC4-tail	66	24.67	47.67	0.81	0.9
TGFB1I1_LD-tat & tat-CAST_1	65.67	70.67	68	0.97	0.06
TGFB1I1_LD-tat & tat-ACTN1_VBS	65.67	68.67	69.67	0.99	0.67
pal-SDC4_middle & pal-SDC4_middle	1.67	24.67	15	0.99	0.41
pal-SDC4_middle & TGFB1I1_LD-tat	1.67	65.67	64.67	0.99	0.41
pal-SDC4_middle & tat-CAST_1	1.67	70.67	64.33	0.99	0.05*
pal-SDC4_middle & tat-ACTN1_VBS	1.67	68.67	65.33	0.99	0.09
pal-SDC4_middle & pal-KK-SDC4-tail	1.67	3	3	0.97	0.6
pal-ITGB3_middle1 & TGFB1I1_LD-tat	43.67	65.67	65.33	0.94	0.68
pal-ITGB3_middle1 & pal-SDC4_middle	43.67	1.67	4.67	0.1	0.99
pal-ITGB3_middle1 & pal-SDC4_JM	43.67	64.33	49	0.8	0.5
pal-ITGB3_middle1 & tat-CAST_1	43.67	70.67	68.33	0.95	0.41
pal-ITGB3_middle1 & tat-ACTN1_VBS	43.67	68.67	68	0.96	0.5
pal-ITGB3_middle1 & pal-KK-SDC4-tail	43.67	3	14	0.2	0.82
pal-ITGB3_middle1 & pal-SDC4-tail	43.67	24.67	40.67	0.5	0.8
pal-SDC4_JM & TGFB1I1_LD-tat	64.33	65.67	66	0.87	0.5
pal-SDC4_JM & pal-SDC4_middle	64.33	1.67	20	0.04*	0.97
pal-SDC4_JM & tat-CAST_1	64.33	70.67	67.67	0.96	0.06
pal-SDC4_JM & tat-ACTN1_VBS	64.33	68.67	68.67	0.91	0.41
pal-SDC4_JM & pal-KK-SDC4-tail	64.33	3	29.67	0.33	0.99
pal-SDC4_JM & pal-SDC4-tail	64.33	24.67	23	0.08	0.32
tat-ACTN1_VBS & tat-CAST_1	68.67	70.67	69.33	0.7	0.32
pal-ITGA2B_JM & pal-ITGA2B_tail	49.33	66	42.67	0.5	0.04*
pal-ITGA2B_JM & TGFB1I1_LD-tat	49.33	65.67	68	1	0.97
pal-ITGA2B_JM & pal-SDC4_middle	49.33	1.67	4	0.05*	0.97
pal-ITGA2B_JM & pal-ITGB3_middle1	49.33	43.67	51	0.65	0.5
pal-ITGA2B_JM & pal-SDC4_JM	49.33	64.33	21.67	0.25	0.04*
pal-ITGA2B_JM & tat-CAST_1	49.33	70.67	70	1	0.5
pal-ITGA2B_JM & tat-ACTN1_VBS	49.33	68.67	62	0.94	0.13
pal-ITGA2B_JM & pal-KK-SDC4-tail	49.33	3	10.33	0.05*	0.99
pal-ITGA2B_JM & pal-SDC4-tail	49.33	24.67	4.33	0.05*	0.41
Pal-KK-SDC4-tail & tat-CAST_1	3	70.67	67.33	0.99	0.13
Pal-KK-SDC4-tail & tat-ACTN1_VBS	3	68.67	67.67	0.99	0.25

Pal-KK-SDC4-tail & TGFB1I1_LD-tat	3	65.67	64.33	0.99	0.32
pal-SDC4-tail & TGFB1I1_LD-tat	24.67	65.67	63	0.9	0.33
Pal-SDC4-tail & Tat-CAST_1	24.67	70.67	44	0.8	0.05*
Pal-SDC4-tail & tat-ACTN1_VBS	24.67	68.67	65.33	0.95	0.32
Pal-SDC4-tail & Pal-KK-SDC4-tail	24.67	3	25	0.5	0.91

S5 Table: Inhibitory effects of peptide combinations on TRAP activation of platelets: comparison with peptides at double concentrations. Combination data is as per Table S4, but is contrasted here with double concentrations of each individual peptide**.** * P<=0.05, two-tailed t-test, as in Table S4.
